# Irradiation effects in monazite–(Ce) and zircon: Raman and photoluminescence study of Au-irradiated FIB foils

**DOI:** 10.1007/s00269-018-0975-9

**Published:** 2018-05-23

**Authors:** Lutz Nasdala, Shavkat Akhmadaliev, Andreas Artac, Chutimun Chanmuang N., Gerlinde Habler, Christoph Lenz

**Affiliations:** 1 Institut für Mineralogie und Kristallographie, Universität Wien, Althanstr. 14, 1090 Vienna, Austria; 2 Institut für Ionenstrahlphysik und Materialforschung, Helmholtz-Zentrum Dresden-Rossendorf e.V., Bautzner Landstr. 400, 01328 Dresden, Germany; 3 Present Address: Sandoz GmbH, Biochemiestr. 10, 6250 Kundl, Austria; 4 Department für Lithosphärenforschung, Universität Wien, Althanstr. 14, 1090 Vienna, Austria; 5 Present Address: Institute of Materials Engineering, Australian Nuclear Science and Technology Organisation, Lucas Heights, NSW 2234 Australia

**Keywords:** Radiation damage, Heavy-ion irradiation, Focused ion beam, Raman spectroscopy, Photoluminescence

## Abstract

Lamellae of 1.5 µm thickness, prepared from well-crystallised monazite–(Ce) and zircon samples using the focused-ion-beam technique, were subjected to triple irradiation with 1 MeV Au^+^ ions (15.6% of the respective total fluence), 4 MeV Au^2+^ ions (21.9%) and 10 MeV Au^3+^ ions (62.5%). Total irradiation fluences were varied in the range 4.5 × 10^12^ – 1.2 × 10^14^ ions/cm^2^. The highest fluence resulted in amorphisation of both minerals; all other irradiations (i.e. up to 4.5 × 10^13^ ions/cm^2^) resulted in moderate to severe damage. Lamellae were subjected to Raman and laser-induced photoluminescence analysis, in order to provide a means of quantifying irradiation effects using these two micro-spectroscopy techniques. Based on extensive Monte Carlo calculations and subsequent defect-density estimates, irradiation-induced spectroscopic changes are compared with those of naturally self-irradiated samples. The finding that ion irradiation of monazite–(Ce) may cause severe damage or even amorphisation, is in apparent contrast to the general observation that naturally self-irradiated monazite–(Ce) does not become metamict (i.e. irradiation-amorphised), in spite of high self-irradiation doses. This is predominantly assigned to the continuous low-temperature damage annealing undergone by this mineral; other possible causes are discussed. According to cautious estimates, monazite–(Ce) samples of Mesoproterozoic to Cretaceous ages have stored only about 1% of the total damage experienced. In contrast, damage in ion-irradiated and naturally self-irradiated zircon is on the same order; reasons for the observed slight differences are discussed. We may assess that in zircon, alpha decays create significantly less than 10^3^ Frenkel-type defect pairs per event, which is much lower than previous estimates. Amorphisation occurs at defect densities of about 0.10 dpa (displacements per lattice atom).

**Electronic supplementary material:**

The online version of this article (10.1007/s00269-018-0975-9) contains supplementary material, which is available to authorized users.

## Introduction

Minerals that incorporate the radionuclides U and Th in their lattice may suffer severe structural damage due to the long-term impact of natural radioactivity. The irradiation damage is created predominantly in alpha-decay events, and here mainly by the nuclear interaction (i.e. atomic “knock-ons”) of heavy daughter nuclei with lattice atoms, caused by their recoil impulse upon emission of an alpha particle. The action of these recoil nuclei, including further atomic displacements caused by displaced lattice atoms, results in the formation of nanometre-sized defect clusters (Wasiliewski et al. 1973; Weber et al. 1994a; Devanathan et al. 2006). With progressive damage accumulation, i.e. if no significant thermal annealing takes place (Nasdala et al. 2001), some minerals eventually may be transformed to a so-called “metamict” state (Ewing 1994). An example for this is the mineral zircon (ZrSiO_4_; tetragonal space group *I*4_1_/*amd*), which often is observed in a genuinely metamict (i.e. irradiation-amorphised) state (Capitani et al. 2000; Nasdala et al. 2002; Zamyatin et al. 2017). A somewhat contrasting example is the mineral monazite–(Ce) [CePO_4_; monoclinic space group *P*2_1_/*n*; the mineral nomenclature follows Lewinson (1966) and Bayliss and Levinson (1988)] that typically shows rather moderate radiation damage in spite of significant Th concentrations (Seydoux-Guillaume et al. 2007; Ruschel et al. 2012). Natural monazite–(Ce) lacks significant defect clusters but is characterised by strong lattice strain and mosaic-like domain texture (that is, volume regions with slightly variable orientation resulting from repeated irradiation and annealing) that is seen in TEM (transmission electron microscopy) dark-field images as “mottled” contrast (Black et al. 1984; Seydoux-Guillaume et al. 2002, 2004; Nasdala et al. 2010c). One main factor controlling of whether or not a mineral species is able to accumulate the self-irradiation damage, or undergoes self-annealing, is the different mineral-specific temperature dependencies of the above two processes. In the case of monazite–(Ce), thermal annealing occurs at comparably low temperatures (Meldrum et al. 1998). Furthermore, it has been proposed, and is discussed controversially since, as to which degree the action of alpha particles, in addition to creating Frenkel-type defects (Nasdala et al. 2011, 2013b), may anneal alpha-recoil damage (Soulet et al. 2001; Gautheron et al. 2009; Deschanels et al. 2014; Li et al. 2017). Such alpha-assisted annealing, if relevant for a certain mineral species, might explain why actinide-bearing minerals do not accumulate radiation damage even at low temperatures.

The accumulation of radiation damage is associated with changes of the host mineral’s physical properties and a general decrease of its chemical durability. This includes, for instance, the increased susceptibility of radiation-damaged minerals to chemical alteration (Horie et al. 2006; Lenting et al. 2010; Seydoux-Guillaume et al. 2012) and to the loss of Pb; the latter may strongly bias the results of isotopic age determinations (Goncalves et al. 2005; Kuiper 2005; Nasdala et al. 2010b). Further, radiation-damaged minerals, and their synthetic analogues, have become important objects in materials-science research, stimulated by the potential use of mineral-like ceramics as host forms for the immobilisation of spent nuclear fuel and other radioactive waste (Omelyanenko et al. 2007; Weber et al. 2009; Montel 2011; Ewing and Weber 2011). For more sound interpretations of the post-growth history of minerals, avoidance of biased isotope age results, as well as for the performance assessment of mineral-like host ceramics, improved quantitative knowledge of radiation effects in minerals is needed.

In recent years, confocal spectroscopic techniques with laser excitation, including Raman (e.g., Guenthner et al. 2013; Wang et al. 2014; Švecová et al. 2016; Marillo-Sialer et al. 2016; Baughman et al. 2017; Váczi and Nasdala 2017; Zietlow et al. 2017) and photoluminescence (PL; e.g., Panczer et al. 2012; Nasdala et al. 2013b; Shimizu and Ogasawara 2014; Lenz and Nasdala 2015), are applied increasingly to estimate the degree of irradiation damage in minerals. The use of these techniques is favoured by a number of analytical advantages, including the opportunity to perform analyses non-destructively and without the need for special sample preparation, and a spatial resolution on the µm^3^ range. A disadvantage, however, is that results often remain “semi-quantitative”, as the correlation between spectral changes and the causal degree of alpha-event damage is still unknown. The possible calibration of radiation-damage-induced spectral changes by reference analyses of well-characterised, naturally radiation-damaged minerals, however, is intricate because of the unknown thermal history, and hence uncertain potential annealing experienced by geological samples.

The latter problem may be overcome by obtaining spectra of (i) initially non-damaged minerals that were ion-irradiated in the laboratory under controlled conditions (e.g. Picot et al. 2008; Zhang et al. 2008; Nasdala et al. 2010a, 2013b; Deschanels et al. 2014; Li et al. 2017) or (ii) synthetic samples doped with short-lived alpha emitters such as ^238^Pu, ^241^Am or ^244^Cm (e.g. Luo and Liu 2001; Burakov et al. 2004, 2010, and references therein) Bregiroux et al. 2007; Deschanels et al. 2014; Shiryaev et al. 2016; see also). In the present study, we have attempted to contribute to the quantitative understanding of heavy-ion damage in accessory minerals by subjecting zircon and monazite–(Ce) samples to irradiation with Au ions of 1–10 MeV energy. These Au energies are about one to two orders of magnitude higher than energies of heavy daughter nuclei in natural alpha-decay events (0.06–0.16 MeV). Gold ions with MeV energy were chosen because their penetration depths in minerals of up to 2 µm allows to irradiation-damage sample volumes that are analysable with modern confocal spectrometer systems. In contrast, irradiation with heavy ions whose energies are in the 0.06–0.16 MeV range would result in damaged layers of ≤ 0.05 µm thickness only. Analysing such shallow volumes requires the application of high-resolution techniques such as Rutherford backscattering spectrometry (Grambole et al. 2007) or transmission electron microscopy (Lian et al. 2002). The main objective of the present study, however, is to contribute to our understanding of irradiation effects in minerals as detected by Raman and PL spectroscopy, for the examples of zircon and monazite–(Ce), which obliged us to choose higher ion energies. The results obtained from our heavy-ion-irradiated samples are compared with the analogous spectral changes of naturally radiation-damaged geological samples, based on displacement numbers as predicted by Monte Carlo simulations.

## Methodological aspects

A crucial aspect of the present irradiation study, following the approach of Nasdala et al. (2010a), is that thin foils instead of bulk samples were irradiated. The irradiation of bulk samples with MeV Au ions, in contrast, would have resulted in the formation of a shallow radiation-damaged layer on top of a non-irradiated and hence still crystalline host. This would be problematic for two reasons. First, damage in the surficial, irradiated layer must result in volume expansion; however, expansion is hindered by the adjacent (non-irradiated and hence non-expanded) host, resulting in compressive strain in the former and tensile strain in the latter. The strain, in turn, strongly affects vibrational modes and crystal-field effects in the damaged layer and hence must bias Raman and PL spectroscopic results. Second, spectroscopic micro-analyses, even if done with state-of-the-art confocal systems, have a depth resolution that is limited to ca. 4λ/(NA)^2^ (with *λ* = excitation wavelength and NA = numerical aperture of the objective used; see Baldwin and Batchelder 2001; Everall et al. 2007). When analysing a µm-thin, irradiation-damaged layer on top of an non-irradiated host crystal (e.g. Zhang et al. 2008; Picot et al. 2008; Mendoza 2010), the signal most probably stems from both the damaged layer and the underlying crystalline host (discussed in detail in Nasdala et al. 2010a; see also; Everall 2008). This is particularly problematic insofar as Raman and luminescence signals of severely damaged solids typically are low in intensity; the signal of the thin damaged layer therefore is obscured easily by the spectrum of the underlying host.

The problem above is avoided by using thin lamellae or foils whose thicknesses correspond to the penetration depths of the ions irradiated. Irradiating bulk samples and removing the non-irradiated host afterwards, in contrast, seems less expedient, as mechanical or other removal of the non-irradiated host may bias analytical results by causing changes to the irradiated layer. Only the irradiation of thin samples that were prepared prior to the irradiation experiment allows analysing the very same sample before and after ion irradiation.

On the one hand, foils or lamellae to be irradiated should be as thin as possible to enable experiments with moderate ion energies (note that the thicker the material, the higher the ion energy needed to create damage throughout the sample). On the other hand, the foils to be irradiated should be significantly thicker than the laser wavelength used for spectroscopy, to avoid band broadening due to surface-strain and other disturbing effects (compare Salje 1973). Nasdala et al. (2010a) have observed that the Raman spectrum of 1 µm thick FIB lamellae of CePO_4_ showed some band broadening, compared to the spectrum of the bulk host, which might be due to surface-strain effects of too thin foils. To account for both aspects, we have chosen sample thicknesses of 1.5 µm in the present study.

## Samples, preparation, irradiation and analyses

### Samples and preparation

The present study was performed on four sets of thin lamellae. These were prepared from one zircon (R–5) and three monazite–(Ce) crystals (Nd3, GM2, N22). The motivation for including not only one but three chemically different monazite–(Ce) samples was founded by the much more variable chemical composition of this mineral, compared to natural zircon (Watt 1995; Williams et al. 2007; Ruschel et al. 2012). We have therefore selected a synthetic CePO_4_ crystal (Nd3), and two natural monazite–(Ce) specimens (GM2, N22) with different contents of non-formula chemical constituents. Origins, ages, and general properties of all four samples are summarized in the electronic supplementary material.

Prior to preparation for ion irradiation, the two natural monazite–(Ce) samples were subjected to dry heat-treatment for 96 h at 1200 °C, to anneal the natural self-irradiation damage and hence to reconstitute samples’ structural state. This temperature and especially the long duration were chosen following Ruschel et al. (2012), to ensure nearly complete annealing of the radiation damage. It is well known that heating of radiation-damaged minerals for a few hours only may not be long enough to ensure (near) equilibrium conditions (Seydoux-Guillaume et al. 2002). For the same reason, zircon sample R–5 was heat-treated for 96 h at 1400 °C. Samples were placed in a Pt crucible and heated at a rate of 10 °C/min to the designated temperature. After the 4-day run, the furnace was shut off and samples remained inside the furnace for another 12 h before the furnace door was opened; samples had cooled down slowly to < 100 °C by then. Slow heating and cooling rates were chosen to avoid any possible bias due to shock heating or quenching. To underline this with an example, Finch et al. (2001) had intentionally “shocked” synthetic pure ZrSiO_4_ crystals by immersing them in liquid N_2_. These authors have obtained unit-cell constants that are significantly larger than that of unquenched ZrSiO_4_ (e.g. van Westrenen et al. 2004), which we assign to a possible quenching-induced build-up of strain.

**Fig. 1:**
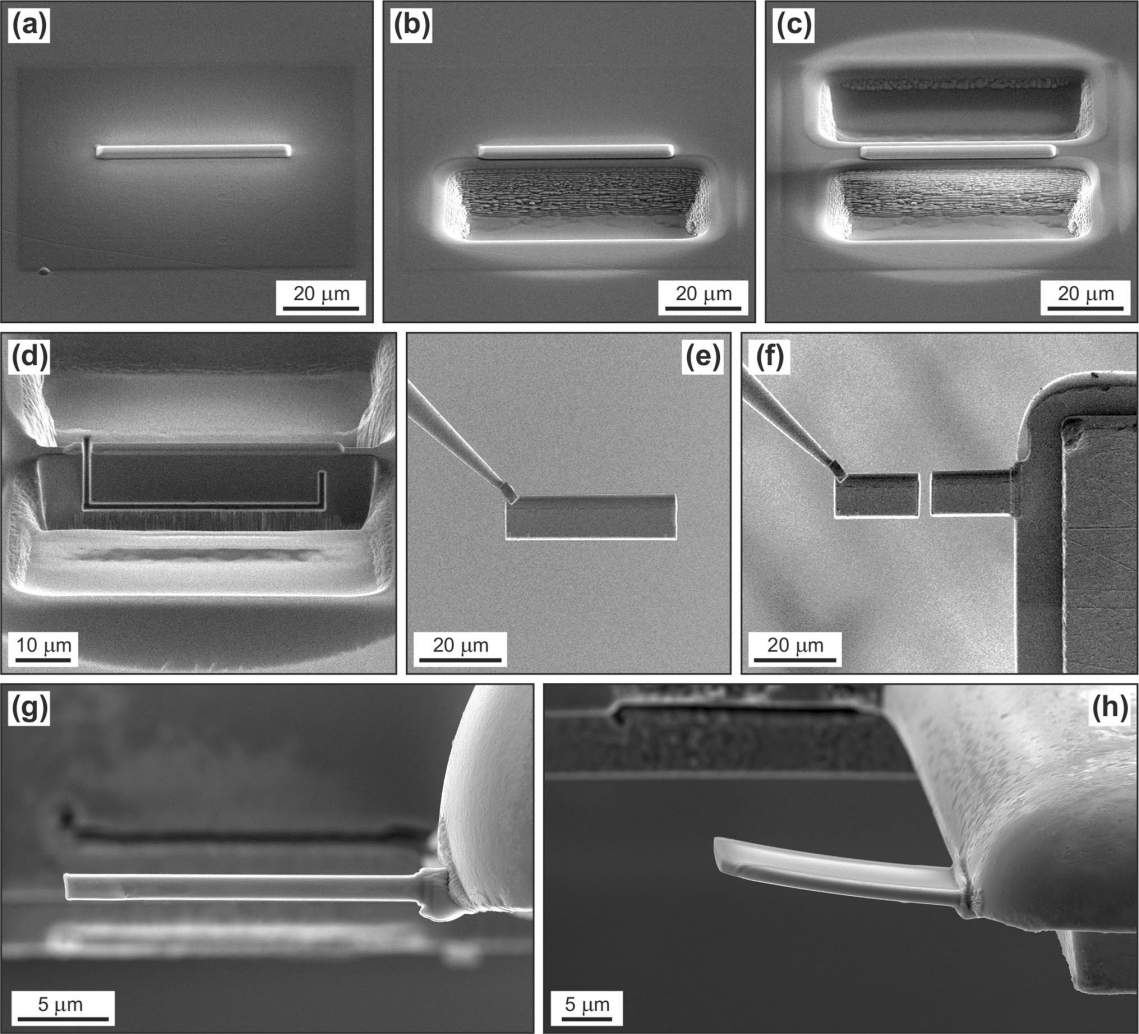
Series of SE images visualizing the FIB-lamella-preparation procedure. **a** Sample surface; the location of future lamellae is marked by Pt deposition at the surface. **b, c** Two cuboid-shaped trenches are dug with a focused Ga beam. There remains a thin “wall” of sample material behind the protective Pt stripe. **d** Sample after tilting around the image’s horizontal axis (approximately 54 ± 3°). The lamella has already been detached incompletely (Ga-beam milling). **e** Lamella being attached to a nanomanipulator tip. **f** Lamella after being cut in half. The right half is attached (Pt deposition) to a Cu post of an Omniprobe lift-out grid, the left half is still attached to the micromanipulator and will be attached to another grid. **g** Lamella after final, low-energy Ga-beam polish, now being plane-parallel. **h** Another lamella after high-fluence Au irradiation. Note the slight bending, which indicates some internal stress gradient

For focused-ion-beam (FIB) preparation of the lamellae to be irradiated, samples were embedded in epoxy, ground, and polished. Then, polished sample surfaces were subjected to chemo-mechanical re-polishing with an alkaline colloidal silica suspension on a polyurethane plate, to remove potential near-surface defects and/or stress in the sample. Surfaces were then coated with carbon. Thin lamellae of cuboid shape, with average sizes of ca. 17–23 × 10–15 µm, and thicknesses of 1.50 ± 0.05 µm, were prepared by means of an FEI Quanta 3D FEG dual beam scanning electron microscope (SEM) equipped with a field-emission Ga source, Pt and C gas-injection systems, and an Omniprobe 100.7 micromanipulator. During foil preparation, the ion beam current was varied in the range 65–1 nA for rough cutting, and 500–300 pA for lamella thinning. The accelerating voltage was set to 30 kV throughout the sputtering and gas deposition procedure. Platinum deposition was used for mechanical stabilization, attaching the cut lamella to a micromanipulator needle for lamella extraction, and final attachment of the lamella to an Omniprobe lift-out grid (Cu). All lamellae belonging to the same sample (R–5, nine lamellae; Nd3, N22, five lamellae each; GM2, four lamellae) were cut at the same orientation relative to the respective host (i.e. parallel with each other). The principal steps of the FIB preparation are visualized in Fig. 1. Note that for enhanced time efficiency, a new FIB preparation protocol was chosen: two lamellae were produced in the course of one cutting and lift-out process.

### Irradiation simulations and experiments

**Fig. 2:**
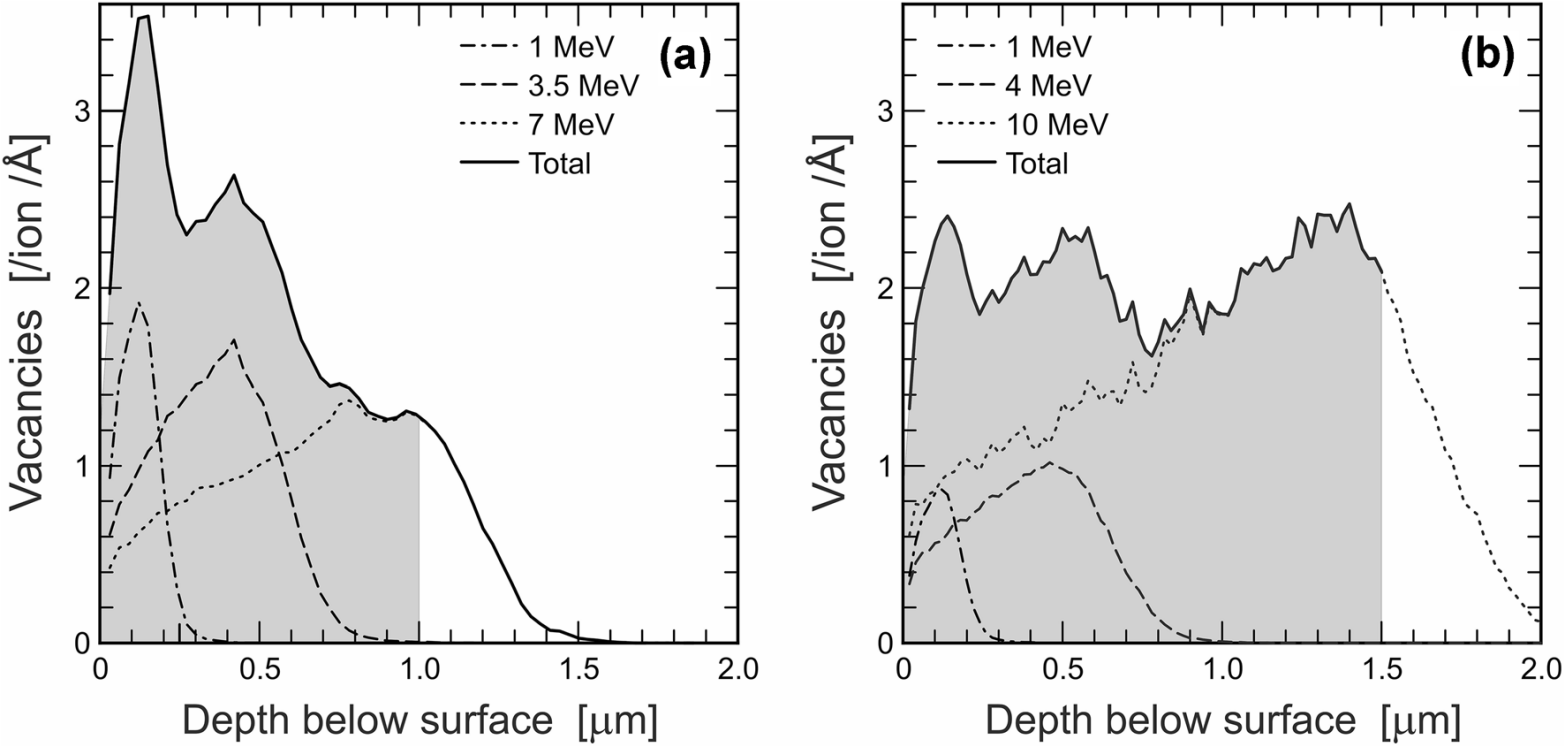
Depth distribution of vacancies in triply Au-irradiated CePO_4_, as predicted by Monte Carlo simulation using SRIM (Ziegler et al. 1985, 2010). Depth-distribution curves are shown for the three individual Au energies and the resulting total damage. Calculations were done using SRIM defaults for displacement energies (O 28 eV, P 25 eV, Ce 25 eV). **a** Defects created by irradiation with 1 MeV (33^1^/_3_ %), 3.5 MeV (33^1^/_3_ %), and 7 MeV (33^1^/_3_ %) Au ions (irradiation protocol of Picot et al. 2008; image modified from Nasdala et al. 2010). Total damage created in a 1 µm thick target is underlain grey. **b** Analogous plot for the modified triple irradiation with 1 MeV (15.6%), 4 MeV (21.9%), and 10 MeV (62.5%) Au ions. Note that a thicker layer (1.5 µm; underlain grey) is damaged, and the damage is predicted to be distributed more homogeneously

We have followed the principal approach of Picot et al. (2008), Nasdala et al. (2010a) and Deschanels et al. (2014) to create damage in a micrometre-sized depth range of the sample by threefold irradiation with Au ions having different energies. In these studies, however, irradiation with equal fluences of 1, 3.5 and 7 MeV Au ions were done. According to Monte Carlo simulations of defect distributions using the SRIM (the Stopping and Range of Ions in Matter; Ziegler et al. 1985, 2010) computer code by Nasdala et al. (2010a), the above irradiation protocol must have resulted in a fairly significant damage-density variation of as much as ± 50% across a 1 µm depth range in CePO_4_ (Fig. 2a).

**Fig. 3:**
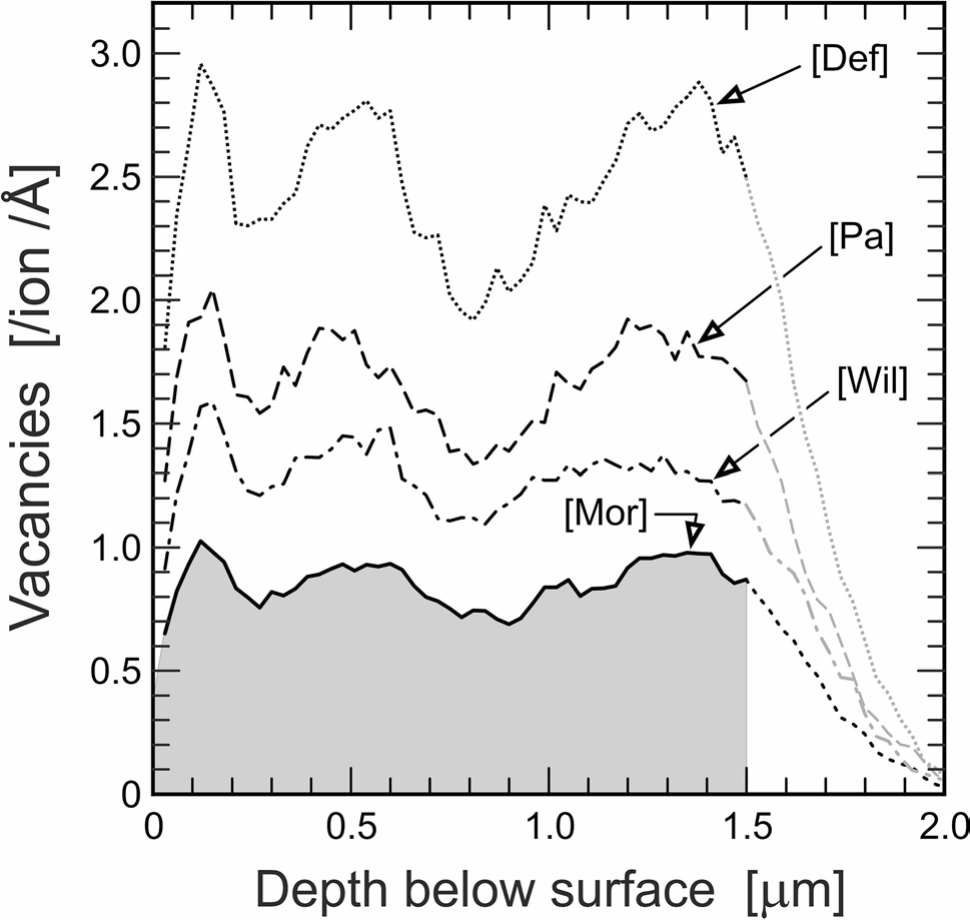
SRIM prediction of the vacancy distribution in a ZrSiO_4_ target, caused by triple Au irradiation analogous to Fig. 2b. Four calculations of the total defect distribution, using four different sets of displacement energies were performed: Def = SRIM defaults (O 28 eV, Si 15 eV, Zr 25 eV); Wil = values of Williford et al. (1998; O 45 eV, Si 20 eV, Zr 80 eV); Pa = values of Park et al. (2001; O 28 eV, Si 48 eV, Zr 89 eV); Mor = values of Moreira et al. (2009; O 60 eV, Si 75 eV, Zr 75 eV). The 1.5 µm target is damaged homogeneously (underlain grey); however, the quantity of vacancies calculated depends strongly on the displacement energies used

In the present study, we have subjected 1.5 µm thick lamellae to triple Au irradiation. In order to find an Au-irradiation protocol that results in less heterogeneously distributed damage in a ∼1.5 µm depth range, we have done systematic SRIM calculations for various Au energies for CePO_4_ and ZrSiO_4_. To the best of our knowledge, no suggested displacement energies for CePO_4_ have been published so far. We have, therefore, used the SRIM defaults (SRIM code version 2013) for displacement energies (Ce, 25 eV; P, 25 eV; O 28 eV). For zircon, calculations were done using the displacement energies of Moreira et al. (2009: Zr, 75 eV; Si 75 eV; O, 60 eV), which are much higher than the SRIM defaults (Zr, 25 eV; Si, 15 eV; O 28 eV). Target densities were assumed as 5.15 g/cm^3^ (CePO_4_) and 4.65 g/cm^3^ (ZrSiO_4_), respectively, and 3000 incoming Au ions were calculated. For practical reasons, we have limited the number of ion energies to be combined (and hence the number of successive irradiations to be made) to three. Also, we attempted to find one set of Au energies and relative fluences that is suitable for the creation of widely homogeneous damage in both CePO_4_ and ZrSiO_4_ (as samples of both minerals were to be irradiated simultaneously). According to the simulation results, triple irradiation of samples with 1 MeV Au^+^ ions (15.6% of the total fluence), 4 MeV Au^2+^ ions (21.9%) and 10 MeV Au^3+^ ions (62.5%) is predicted to result in damage with relative variations of less than ±20% across a 1.5 µm depth range (Figs. 2b, 3).

The triple Au irradiations were done by means of the 3 MV Tandetron accelerator of the Helmholtz-Zentrum Dresden-Rossendorf, Germany. Samples were irradiated in the standard implantation chamber, which was evacuated to ~ 3 × 10^− 7^ mbar. In view of possible sample heating during irradiation, and to diminish immediate defect recombination at elevated temperatures, samples were cooled with liquid N_2_. The beam current was 45–50 nA, resulting in a current density of 11.3–12.5 nA/cm^2^. Based on the results of Nasdala et al. (2010a), the total fluences for triple irradiations done in the present study were varied between 4.5 × 10^12^ and 1.2 × 10^14^ ions/cm^2^.

### Analytical techniques

The general chemical composition of samples used for FIB lamellae preparation was determined by wavelength-dispersive X-ray analysis using a Cameca SX100 electron probe micro-analyser (EPMA). The system was operated at 15 kV, and the beam current was 20 nA [monazite–(Ce)] and 40 nA (zircon), respectively. To avoid analytical bias by the high energy density of a fully-focused beam (which could possibly result, for example, in loss of Na from phosphates), the electron beam was defocused to a fairly large circular area of 8 µm diameter. The following natural and synthetic reference materials were used for calibration: Na, albite; Si, sanidine; P, LaPO_4_; Ca and Th, CaTh(PO_4_)_2_; Y, YAG; Zr, ZrSiO_4_; REEs, individually doped REEPO_3_; Hf, HfSiO_4_; Pb, PbSe; U, U metal. Thorough wavelength-dispersive angle scans were made for choosing reliable background positions, and to check for possible peak overlaps. Peak counting times were 20 s for major elements and 40 s for minor and trace elements; except for Pb (180 s; Pb–Mα line), Th (60 s; Th–Mα line), and U (100 s, U–Mβ line). All background-counting times were set to half of the respective peak counting-time. For more details see Škoda et al. (2015).

Raman and PL measurements were carried out by means of a Horiba LabRAM HR Evolution system equipped with Olympus BX41 optical microscope and Si-based, Peltier-cooled charge-coupled device (CCD) detector. An Olympus 100 × objective (numerical aperture 0.9) was used to focus the light onto the surface of the lamella (focal-spot diameter well below 1 µm). Raman spectra were obtained using He-Ne 632.8 nm excitation (10 mW at the sample surface). The Raman-scattered light was dispersed using a diffraction grating with 1800 grooves per millimetre in the optical pathway, resulting in an instrumental profile function (IPF; also commonly referred to as apparatus function or the spectral resolution performance) of 0.8 cm^− 1^. The PL spectra were obtained with 473 nm (5 mW; for green Dy^3+^ emissions) and 532 nm excitation [12 mW; for Nd^3+^ emissions in the near-infrared (NIR) range], respectively. For PL, a diffraction grating with 600 grooves per millimetre was placed in the optical pathway, resulting in an IPF between ∼ 4 cm^− 1^ (green spectral range) and ∼ 2 cm^− 1^ (NIR spectral range). Wavenumber calibration was done using the Rayleigh line (Raman) and Kr-lamp emissions (PL), respectively. The wavenumber accuracy was better than 0.5 cm^− 1^. Multiple measurements were placed across each lamella, to check for possible lateral variations.

Band and line fitting was done after background subtraction, assuming Lorentzian-Gaussian (i.e. pseudo-Voigt) shapes. All measured signals consist of an overlap of the (predominantly Lorentzian) Raman band or PL line, and the (predominantly Gaussian) IPF, which, as an analytical artefact, results in artificial broadening of the spectroscopic signal (Nasdala et al. 2001; Presser 2009). Measured FWHMs therefore were corrected mathematically for the IPF, and true FWHMs were calculated, using the empirical correction formula of Váczi (2014):

1 $${\text{FWH}}{{\text{M}}_{{\text{true}}}} \approx {\text{FWH}}{{\text{M}}_{{\text{meas}}}} - \frac{{{{({\text{FWH}}{{\text{M}}_{{\text{IPF}}}})}^2}}}{{0.9{\text{FWH}}{{\text{M}}_{{\text{meas}}}}+0.1{\text{FWH}}{{\text{M}}_{{\text{IPF}}}}}}.$$

This formula was preferred over the commonly used correction equation of Dijkman and van der Maas (1976). The latter assumes a triangular IPF and provides reliable estimates only if the IPF contribution is small compared to the true FWHMs, whereas its use tends to result in an “overcorrection” for narrow FWHMs (in detail demonstrated by Presser and Glotzbach 2009). Total uncertainties of the true FWHMs quoted (including IPF correction and lateral variations across the lamellae) are assessed to be smaller than 10%.

## Results

### Analytical characterisation of lamellae

The chemical compositions of the four samples studied (determined prior to FIB preparation and ion irradiation) are summarised in Table 1. Age data for the raw samples, unit-cell constants and selected Raman and PL spectroscopic parameters are listed in the electronic supplementary material (Tables S1–S3). Note that we have not observed any indication for two-phase samples [for zircon reported by Murakami et al. (1991); for monazite–(Ce) reported by Seydoux-Guillaume et al. (2002)] in our X-ray diffraction patterns; therefore only one set unit-cell parameters per sample is quoted in Table S3.

**Table 1:** Chemical compositions of the samples studied (EPMA results; all values in wt%)

Sample (origin)	SiO_2_	P_2_O_5_	CaO	Y_2_O_3_	ZrO_2_	La_2_O_3_	Ce_2_O_3_	Pr_2_O_3_	Nd_2_O_3_	Sm_2_O_3_
*Monazite–(Ce)*										
Nd3 (synthetic)^a^	n.d	30.4	n.d	n.d	n.d	n.d	69.3	n.d	0.24	n.d
GM2 (Itambé)^b^	0.47	29.7	1.18	2.14	n.d	10.5	27.7	3.23	10.5	3.35
N22 (Madagascar)	2.83	26.6	0.15	2.12	n.d	7.26	24.2	3.52	14.8	4.37
*Zircon*										
R–5 (Ratanakiri)	32.4	n.d	n.d	0.02	66.8	n.d	n.d	n.d	n.d	n.d

Sample (origin)	Gd_2_O_3_	Dy_2_O_3_	Ho_2_O_3_	Yb_2_O_3_	HfO_2_	PbO	ThO_2_	UO_2_	Total
*Monazite–(Ce)*									
Nd3 (synthetic)^a^	n.d	n.d	n.d	n.d	n.d	n.d	n.d	n.d	99.9
GM2 (Itambé)^b^	2.22	1.14	n.d	n.d	n.d	0.18	6.57	0.68	99.6
N22 (Madagascar)	2.16	0.41	0.03	n.d	n.d	0.30	10.8	0.47	100.1
Zircon									
R–5 (Ratanakiri)	n.d	n.d	n.d	n.d	0.70	n.d	n.d	n.d	99.7

Data are means of ≥ 5 individual analyses, performed on one crystal (Nd3; N22), or means of ≥ 8 individual analyses, performed on several crystals (R–5; GM2), respectively

*n.d*. not detected

^a^For details of the NaPO_3_ flux synthesis technique see Lenz et al. (2013, 2015)

^b^Data for GM2 are from Ruschel et al. (2012)

**Fig. 4:**
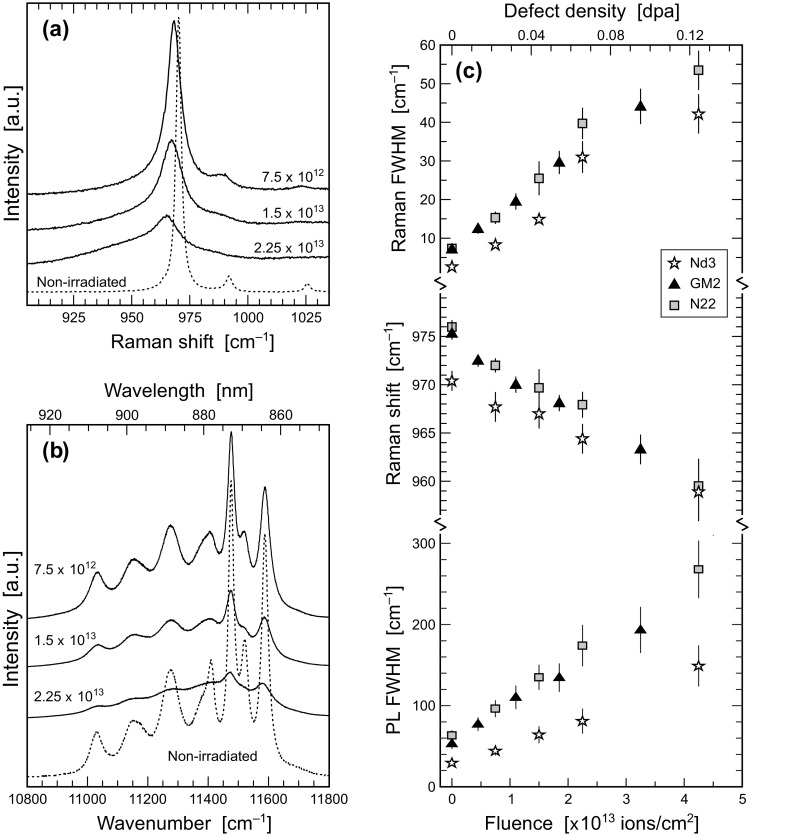
Spectroscopic results obtained from unirradiated and irradiated monazite –(Ce). **a** Selected Raman spectra obtained from sample Nd3 (633 nm excitation). **b** Selected PL spectra (Nd^3+ 4^F_3/2_ → ^4^I_9/2_ transition; 532 nm excitation) obtained from sample Nd3. **c** Plots of the width and spectral position of the ν_1_(PO_4_) Raman band, and the width of the ∼ 11,600 cm^− 1^ Nd^3+^ (^4^F_3/2_ → ^4^I_9/2_) sublevel, of the three samples Nd3, GM2 and N22 against irradiation fluence. The top abscissa quotes fluences converted into defect densities (displacements per lattice atom)

Representative Raman and PL spectra obtained from FIB lamellae before and after irradiation are shown in Figs. 4a, b [monazite–(Ce)] and 5a, b (zircon). Note that all presented Raman and PL spectra were obtained with the same lamellae orientation with respect to laser polarisation. As all lamellae of the same sample were extracted from their respective host in parallel orientation, direct comparison of the spectra is possible without any need to consider possible orientation-induced changes of relative signal intensities. We emphasise that spectroscopic parameters obtained from non-irradiated FIB lamellae (Table 2) are indistinguishable within analytical uncertainties from those obtained from the annealed bulk samples (Supplementary Table S3) used for lamellae preparation. This indicates that the FIB-preparation process itself, and the narrow thicknesses of the lamellae, do not introduce any significant analytical bias.

**Table 2:** Irradiation fluences, calculated defect densities, and spectroscopic parameters of non-irradiated and Au-irradiated FIB lamellae

Sample	Au fluence	Defect density [dpa]	Raman^a^		PL	
			FWHM [cm^− 1^]	Shift [cm^− 1^]	Dy^3+^ FWHM^b^ [cm^− 1^]	Nd^3+^ FWHM^c^ [cm^− 1^]
*Monazite–(Ce)*						
Nd3	Non-irradiated	0.000	2.6 ± 0.3	970.4 ± 1.0	–	29 ± 3
	7.5 × 10^12^	0.022	8.3 ± 0.5	967.7 ± 1.5	–	44 ± 5
	1.5 × 10^13^	0.044	14.9 ± 1.0	967.0 ± 1.5	–	64 ± 7
	2.25 × 10^13^	0.066	31 ± 4	964.4 ± 1.5	–	81 ± 8
	4.5 × 10^13^	0.125	42 ± 5	958.9 ± 3.0	–	149 ± 17
	1.2 × 10^14^	0.353	n.d	n.d	–	n.d
N22	Non-irradiated	0.000	7.3 ± 1.0	976.0 ± 0.7	–	63 ± 6
	7.5 × 10^12^	0.022	15.3 ± 1.5	972.0 ± 0.7	–	96 ± 10
	1.5 × 10^13^	0.044	25.5 ± 4.5	969.7 ± 1.9	–	135 ± 15
	2.25 × 10^13^	0.066	40 ± 4	967.9 ± 1.3	–	174 ± 19
	4.5 × 10^13^	0.125	53 ± 5	959.5 ± 2.8	–	268 ± 30
	1.2 × 10^14^	0.353	n.d	n.d	–	n.d
GM2	Non-irradiated	0.000	7.1 ± 1.0	975.3 ± 0.6	–	53 ± 5
	4.5 × 10^12^	0.012	12.4 ± 1.2	972.5 ± 0.6	–	77 ± 8
	1.1 × 10^13^	0.032	19.5 ± 2.0	970.0 ± 0.8	–	110 ± 12
	1.85 × 10^13^	0.051	30 ± 3	968.1 ± 0.8	–	135 ± 14
	3.25 × 10^13^	0.095	44 ± 5	963.3 ± 1.5	–	193 ± 22
*Zircon*						
R–5	Non-irradiated	0.000	1.8 ± 0.2	1007.6 ± 0.5	13 ± 1	15 ± 2
	4.5 × 10^12^	0.004	5.0 ± 0.5	1005.2 ± 0.5	24 ± 2	n.a
	7.5 × 10^12^	0.007	7.1 ± 0.6	1003.2 ± 0.5	n.a	20 ± 2
	1.1 × 10^13^	0.010	10.9 ± 0.8	1001.9 ± 0.5	40 ± 4	29 ± 3
	1.5 × 10^13^	0.014	13.2 ± 1.1	1000.9 ± 0.5	48 ± 5	32 ± 3
	1.85 × 10^13^	0.017	18.7 ± 1.5	999.3 ± 0.5	67 ± 7	34 ± 4
	2.25 × 10^13^	0.021	20.3 ± 2.3	998.7 ± 0.5	69 ± 7	38 ± 5
	3.25 × 10^13^	0.031	25.8 ± 2.5	996.6 ± 0.5	80 ± 8	39 ± 6
	4.5 × 10^13^	0.040	29.6 ± 3.0	994.5 ± 0.5	n.d	59 ± 11
	1.2 × 10^14^	0.113	n.d	n.d	n.d	n.d

n.a. not analysed

n.d. not detected

^a^Quoted for the ν_1_(PO_4_) [monazite–(Ce)] and the ν_3_(SiO_4_) (zircon) mode, respectively

^b^Quoted for the 17,210 cm^− 1^ sublevel of the ^4^F_9/2_ → ^4^H_13/2_ emission of Dy^3+^

^c^Quoted for the 11,600 cm^− 1^ [monazite–(Ce)] and 11,360 cm^− 1^ (zircon) sublevel of the ^4^F_3/2_ → ^4^I_9/2_ emission of Nd^3+^, respectively

**Fig. 5:**
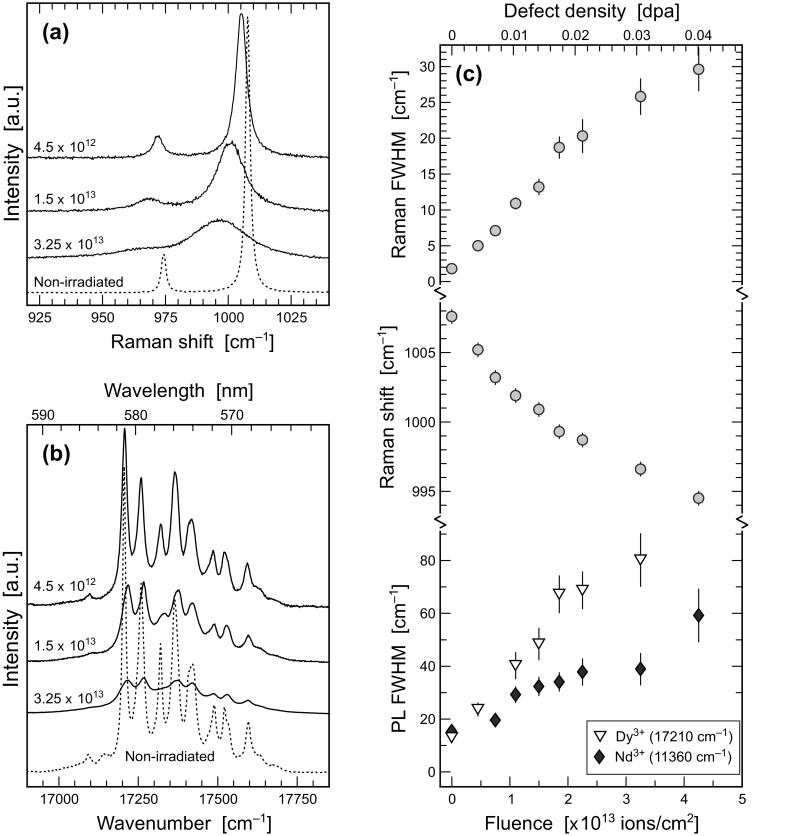
Spectroscopic results obtained from unirradiated and irradiated zircon R–5. **a** Selected Raman spectra (633 nm excitation). **b** Selected PL spectra (Dy^3+ 4^F_9/2_ → ^4^H_13/2_ transition; 473 nm excitation). **c** Plots of the width and spectral position of the ν_3_(SiO_4_) Raman band, and the widths of the ∼ 17,210 cm^− 1^ Dy^3+^ (^4^F_9/2_ → ^4^H_13/2_) sublevel and the ∼11,360 cm^− 1^ Nd^3+^ (^4^F_3/2_ → ^4^I_9/2_) sublevel, against irradiation fluence. The top abscissa quotes fluences converted into defect densities (displacements per lattice atom)

Selected spectroscopic parameters are listed in Table 2 and plotted in Figs. 4c and 5c. Following Nasdala et al. (1995), Seydoux-Guillaume et al. (2002) and Ruschel et al. (2012), we have selected the highest-intensity signals for monitoring irradiation-induced changes in Raman spectra. These are the ν_1_(PO_4_) band of monazite–(Ce) (symmetric stretching of PO_4_ tetrahedrons; A_1_ mode near 970 cm^− 1^; Begun et al. 1981) and the ν_3_(SiO_4_) band of zircon (antisymmetric stretching of SiO_4_ tetrahedrons; B_1_ mode near 1008 cm^− 1^; Dawson et al. 1971). Both of these bands are distinct (i.e. scarcely overlain by other bands) and hence can be fitted with comparably little bias. In PL spectra, one particular Stark line (i.e. sublevel) of the ^4^F_3/2_ → ^4^I_9/2_ electronic transition of Nd^3+^ was chosen (following Lenz et al. 2013). The above transition shows different Stark splitting in the two minerals, due to different crystal-field effects. The ∼ 11,600 cm^− 1^ [monazite–(Ce)] and ∼11,360 cm^− 1^ (zircon) Stark lines, respectively, were found to be most suitable for monitoring irradiation-induced changes. For zircon, the ∼17,210 cm^− 1^ Stark line of the ^4^F_9/2_ → ^4^H_13/2_ emission of Dy^3+^ was used in addition (Lenz and Nasdala 2015). Other Stark lines of the above transitions, and other electronic transitions of Nd^3+^, Dy^3+^ and other emission centres, were found less suitable or even unsuitable for reliably monitoring irradiation-induced spectral changes, as several Stark lines often superimpose each other and thus impede unbiased fitting [elucidated in detail by Lenz and Nasdala (2015) for the example of the Stark splitting of a Sm^3+^ emission].

The principal Raman and PL spectral changes observed from Au-irradiated lamellae (Figs. 4, 5) comprise significant Raman band and PL line broadening, in the case of Raman bands accompanied with shifts toward lower Raman-shift values, and general intensity losses. These spectral changes correspond well to spectral peculiarities of naturally radiation-damaged accessory minerals (e.g. Nasdala et al. 1995, 2001, 2004, 2013b; Zhang et al. 2000; Seydoux-Guillaume et al. 2002; Shimizu and Ogasawara 2014; Lenz and Nasdala 2015; Švecová et al. 2016). The striking difference, however, is that in contrast to the experimentally irradiated monazite–(Ce) lamellae, naturally self-irradiated monazite–(Ce) has, to the best of our knowledge, never been observed in a severely damaged or even fully metamict state (Ruschel et al. 2012). Hence the Raman band and PL line broadening of our lamellae exceed appreciably the damage-induced spectral changes known from natural monazite–(Ce).

The three monazite–(Ce) and zircon lamellae that were irradiated with the highest fluence of 1.2 × 10^14^ Au ions/cm^2^ (Table 2) did not yield any Raman bands of crystalline CePO_4_ or ZrSiO_4_, respectively, anymore. Only a low-intensity, broad hump centred near 950 cm^− 1^ (not shown) was detected, which is assigned to the Raman signal of amorphous CePO_4_ (Nasdala et al. 2010a) and amorphous ZrSiO_4_ (Zhang et al. 2000; Nasdala et al. 2001), respectively. Similarly, PL spectra of these three samples yielded low-intensity, broad emission bands without any noticeable fine structure (not shown). Such emissions are assigned to degenerate electronic transitions as typical of a disordered ligand environment lacking any crystal-field splitting (compare Fig. 1a in Nasdala et al. 2013a).

### Defect densities in irradiated lamellae

For comparison of the results of our irradiation experiments with that of previous publications, and in particular to provide a more independent measure of correlation between the degree of damage and associated certain spectral changes, we have calculated defect densities in the irradiated lamellae. In order to express defect densities we do not use the density of the vacancies created, but rather the average of atomic displacements per lattice atom (dpa). It should be noted that the abbreviation dpa is used inconsistently in the literature. In some cases it represents the number of displacements created per primary atom irradiated whereas in other cases it describes the number of displacements per lattice atom of the target. In the present study, we use dpa only for the latter (i.e. a defect density of 0.10 dpa means that 10% of all target-lattice atoms were displaced from their initial sites).

The conversion of Au-irradiation fluences into dpa was based on defect numbers predicted by Monte Carlo calculations (SRIM code 2013) that have included calculations of full cascades (i.e. also considering sub-branches of displacements caused by displaced lattice atoms). For CePO_4_ the results predict that triple irradiation with 1 MeV Au^+^ (15.6%), 4 MeV Au^2+^ ions (21.9%) and 10 MeV Au^3+^ ions (62.5%) causes on average 36,925 atomic displacements per incoming Au ion in the target (finally creating 36,035 vacancies whereas 890 defects are recombined immediately). However, about 13.3% of the defects are created at locations more than 1.5 µm below the target surface (Fig. 2b). Therefore, 32,016 defects per incoming Au ion are predicted to be created in a 1.5 µm thick lamella. Analogously, an average of 14,780 defects per incoming Au ion are predicted for a ZrSiO_4_ target, of which 13,004 are located in the depth range 0–1.5 µm (Fig. 3). As the two target materials have different chemical compositions and densities, consequently, the same irradiation is expected to result in somewhat different amounts of defects in the two different materials. The significance in difference of the predicted defect numbers in CePO_4_ and ZrSiO_4_ (32,016 versus 13,004), however, is due to the fact that SRIM defaults for atomic displacement energies have been used only for CePO_4_. For ZrSiO_4_, in contrast, we have used the much more realistic displacements energies of Moreira et al. (2009). These energies are much higher than the defaults and, consequently, have resulted in a much lower defect number (Fig. 3). To visualise the strong dependence of the predicted amount of defects on the displacement energies, we have repeated the calculations for ZrSiO_4_ also for the sets of displacement energies proposed by Williford et al. (1998; O 45 eV, Si 20 eV, Zr 80 eV) and Park et al. (2001; O 28 eV, Si 48 eV, Zr 89 eV). As SRIM defaults for displacement energies for atoms in ZrSiO_4_ are significantly underestimated, it appears reasonable to assume the same for CePO_4_. The calculated defect number for CePO_4_ therefore is considered significantly too high. More precise estimates for displacement energies of Ce, P and O in CePO_4_, for instance based on molecular-dynamical calculations, are needed.

Second, the displacement concentration (per cm^3^) in each 1.5 µm lamella irradiated was calculated. For this, the above displacement number per incoming Au ion was multiplied by the respective Au fluence (per cm^2^) and by 10^4^/1.5. The unit-cell volumes of ∼ 300 Å^3^ ([monazite–(Ce)] and ∼ 260 Å^3^ (zircon) convert to ∼8 × 10^22^ atoms per cm^3^ [monazite–(Ce)] and ∼ 9.23 × 10^22^ atoms per cm^3^ (zircon), respectively. The ratio of displacements per cm^3^ and total number of atoms per cm^3^ yields the displacement fraction (dpa). The results of the conversions of fluences into displacements per lattice atom are quoted in Table 2, and they were plotted also on the top abscissa axis in Figs. 4c and 5c. Mildly-to strongly but not yet amorphous lamellae (irradiations with Au fluences between 4.5 × 10^12^ and 4.5 × 10^13^ ions/cm^2^) are characterised by calculated defect densities of 0.004–0.040 dpa (zircon) and 0.012–0.125 dpa [monazite–(Ce)] and amorphised lamellae (1.2 × 10^14^ ions/cm^2^) have calculated defect densities of ∼ 0.11 dpa (zircon) and ∼0.35 dpa [monazite–(Ce)], respectively.

The dpa values for monazite–(Ce), however, are most likely overestimated and hence biased, because they were calculated using too high displacement numbers (as discussed above). We therefore cannot evaluate them quantitatively. For zircon, Raman and PL spectra showed strong band/line broadening and intensity losses at 0.040 dpa, indicating severe irradiation damage. At 0.113 dpa, no remnant crystalline Raman signal or crystal-field splitting was observed, indicating amorphisation. Consequently, amorphisation of zircon, presumably through lattice collapse due to too high vacancy and interstitial concentrations, must take place at a defect density in the range of 0.04–0.11 dpa. This low value is on the same order as amorphisation-critical defect densities of other solids. For instance, amorphisation was described at below 0.10 ± 0.05 dpa in on-irradiated diamond (Lee et al. 1993) and at below ∼0.16 dpa in ^244^Cm-doped Gd_2_Ti_2_O_7_ (Weber et al. 1986; discussed in; Lian et al. 2003).

### Defect densities in natural reference samples

The (at least “semi-quantitative”) comparative evaluation of how damage in the Au-irradiated lamellae on the one hand, and alpha-event damage in naturally self-irradiated samples on the other hand, are related, requires a comparison means. Simply matching fluences and doses is impossible, because of different energies and different species of high-energy ions involved in the two processes, and because of the strong dependence of the ratio of nuclear and electronic stopping powers on the ion mass and energy. We have, therefore, also converted the alpha doses received by well-studied natural minerals into dpa. For this comparison, we have chosen 16 monazite–(Ce) samples of Mesoproterozoic to Cretaceous ages described by Ruschel et al. (2012) plus monazite–(Ce) N22 studied herein, and assumed-to-be non-annealed zircon samples from four localities discussed by Nasdala et al. (2001).

The principal approach of the conversion was analogous to the above. It is described in detail in the online supporting material. First, numbers of defects created in an alpha-decay event were predicted by SRIM calculation. These calculations were done for all alpha energies in the ^238^U, ^235^U and ^232^Th decay series that have a relative probability of ≥ 10% in the particular decay event (cf. Firestone and Shirley 1996). The numbers of displacements per event are calculated as the sums of displacements created by the alpha particle and displacements by the corresponding recoil nucleus. Weighted means of displacements per alpha-event were then calculated for the ^238^U, ^235^U and ^232^Th decay series (electronic supplementary material; Tables S4 and S6). These means were then used to convert time-integrated alpha-doses into dpa (electronic supplementary material; Tables S5 and S7).

For the natural monazite–(Ce) samples, the defect densities calculated from their alpha doses (1.2–10.8 × 10^19^ events/g), based on 1484–1645 defects per alpha event (Table S4), lie in the range 1.28–11.31 dpa (Table S5). Analogous to what we have discussed above for SRIM-based estimates of Au-irradiated monazite–(Ce) foils, these estimates are likely overestimated because of the (probably too low) SRIM defaults for displacements energies. This bias, however, should not significantly affect the comparability of dpa values calculated for Au-irradiated lamellae and self-irradiated natural samples, as both calculations are affected by the same underlying uncertainty. It also needs to be considered that at elevated defect concentrations the probability of re-displacement of already displaced atoms increases; hence the total number of displacement processes must be significantly higher than the number of finally displaced atoms.

For ZrSiO_4_, our SRIM calculations predict 620–688 displacements per alpha-decay event (alpha recoil plus alpha particle damage; Table S6). Based on these means, the alpha doses of naturally self-irradiated zircon samples described by Nasdala et al. (2001) convert to defect densities in the range of 0.002–0.057 dpa (Table S7).

## Discussion

### General characterisation of starting materials

Upon heat treatment prior to the preparation of FIB lamellae, the two natural monazite–(Ce) samples N22 and GM2 have experienced significant decreases of unit-cell dimensions and FWHMs of Raman bands and PL lines, along with upshifts of the spectral position of the ν_1_(PO_4_) Raman band. These parameter changes are assigned to the structural reconstitution through annealing of radiation damage (compare Seydoux-Guillaume et al. 2002; Ruschel et al. 2012). This in turn indicates that the initial samples had accumulated significant amounts of self-irradiation damage, whose presence (i.e. if no annealing was done prior to ion irradiation) might have biased the results of our study. Our observations correlate well with the high self-irradiation doses of 4.43 × 10^19^ α/g (N22) and 3.15 × 10^19^ α/g (GM2), respectively, calculated from U–Pb ages and present U and Th concentrations.

The opposite was observed for zircon sample R–5. X-ray diffraction and spectroscopic parameters of original and annealed samples are identical within errors. This indicates that the Ratanakiri zircon had not accumulated noticeable amounts of radiation damage. This, in turn, is in accordance with the very young U–Pb age of less than 1 Ma (cf. electronic supplementary material; Table S2 and Fig. S2) and low mean concentrations of ∼ 95 ppm Th and ∼ 120 ppm U (unpublished laser ablation-inductively coupled plasma-mass spectrometry results; C. Petautschnig, personal communication). An extremely low self-irradiation dose of 0.4 × 10^15^ α/g is calculated for the Ratanakiri zircon, which is two orders below the minimum self-irradiation level of 0.05–0.1 × 10^18^ α/g that is known to cause minute spectroscopically detectable changes to zircon (Zhang et al. 2000; Nasdala et al. 2004).

Deviations of the X-ray and Raman parameters of the annealed monazite–(Ce) samples N22 and GM2 (supplementary Table S3) from that of synthetic undoped CePO_4_ (Ni et al. 1995; Ruschel et al. 2012) are assigned to the chemical composition of the former, i.e. the presence of significant amounts of non-formula elements in natural monazite–(Ce). Contrastingly, unit-cell constants and spectroscopic parameters of zircon R–5 (supplementary Table S3) are not significantly different from that of synthetic pure ZrSiO_4_ (Nasdala et al. 2002; van Westrenen et al. 2004), which is in accordance with the generally low level of non-formula elements in sample R–5.

### Spectroscopic changes of irradiated lamellae

Our observation of amorphisation between 4.5 × 10^13^ and 1.2 × 10^14^ Au ions/cm^2^ corresponds well to the TEM results of Deschanels et al. (2014) who observed amorphisation of LaPO_4_ between 1.73 × 10^13^ and 7.2 × 10^14^ ions/cm^2^ (triple irradiation with 1, 3.5 and 7 MeV Au ions). It should be noted, however, that degrees of damage detected by Raman spectroscopy on the one hand and TEM techniques and X-ray diffraction on the other hand, may differ appreciably (Chanmuang et al. 2017), and hence amorphisation may be detected at somewhat different fluences with different techniques.

The spectral changes (Raman band broadening and down-shift and PL line broadening) of all other lamellae (Au fluences 4.5 × 10^12^–4.5 × 10^13^ ions/cm^2^) correlate nearly linearly with the Au-irradiation fluence (Figs. 4c, 5c). A slight bend at higher fluences may perhaps point to a hypothetical maximum/saturation level for the change of the respective spectral parameter. Irradiation with the same Au fluences has resulted in similar extent of spectroscopic changes in the two minerals studied, implying similar degrees of irradiation-induced damage. Our irradiation experiments hence contradict again the results of Picot et al. (2008) who proposed PO_4_ tetrahedrons as particularly irradiation-resistant structural units, and confirm the results of Nasdala et al. (2010a) and Deschanels et al. (2014) who found the opposite. Our present results support again that monazite–(Ce) is not a particularly irradiation resistant mineral; rather it has an irradiation response that is quite similar to that of zircon.

There is, however, a systematic quantitative difference between our spectroscopic results and the results of Nasdala et al. (2010a); significantly lesser FWHM increases of Raman bands have been observed in that earlier study. To quote an example, irradiation of synthetic CePO_4_ with a total Au (1–7 MeV) fluence of 1.8 × 10^13^ ions/cm^2^ by Nasdala et al. (2010a) has resulted in a FWHM increase from 4.6 to 8.8 cm^− 1^. In the present study, comparable irradiation of synthetic CePO_4_ (somewhat lower fluence of 1.5 × 10^13^ ions/cm^2^, however, with somewhat higher ion energies in the range 1–10 MeV) has resulted in a much more pronounced FWHM increase from 2.6 to 14.9 cm^− 1^. The systematic difference is assigned to the fact that samples were cooled with liquid N_2_ in the present study only, whereas irradiations by Nasdala et al. (2010a) were done at room temperature. Failure in sample cooling during ion irradiation obviously resulted in a significantly enhanced fraction of immediate defect recombinations, which is supposedly due to (i) the high susceptibility of monazite-structured materials to undergo structural recovery even at comparably low temperatures [low critical amorphisation temperatures *T*_c_ between 60 °C and 175 °C have been reported by Meldrum et al. (1998)], and (ii) sample heating as caused by the high energy density of the ion beam itself.

### Comparison with natural monazite–(Ce) samples

**Fig. 6:**
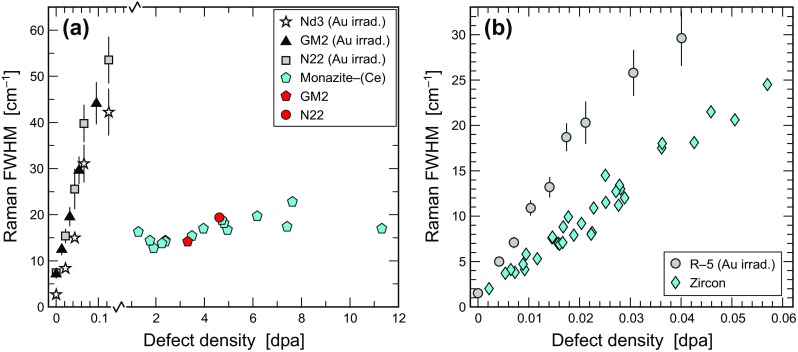
Comparison of the Raman-band broadening of irradiated FIB lamellae (greyscale symbols; data pairs from Figs. 4c, 5c) in comparison with data for self-irradiated natural samples (coloured symbols). **a** Monazite–(Ce) (pentagons, data from Ruschel et al. 2012). Note the vastly different defect-density scaling on the two sides of the x-axis break. **b** Zircon (diamonds, data for “unannealed” samples from Nasdala et al. 2001)

The high defect densities (1.28–11.31 dpa) calculated for natural monazite–(Ce) samples in Table S5 imply amorphisation or at least severe damage. This, however, was not observed. All 17 monazite–(Ce) samples, in spite of huge self-irradiation doses of 12.5–108.0 × 10^18^ α/g, are just mildly to moderately radiation-damaged (Ruschel et al. 2012; Fig. 6a). This is in apparent contrast to our observation that comparably moderate Au irradiation results in severe damage, hence confirming that monazite–(Ce) is not at all an irradiation-resistant phase (Fig. 6a). There are two possible interpretations; perhaps both apply. First, low damage-retention degrees of natural samples must be assigned to a limited long-term stability of irradiation-induced damage in this mineral. This in turn corresponds very well with the much lower temperatures that are needed to anneal monazite–(Ce) compared to zircon (Meldrum et al. 1998). Second, if alpha-assisted annealing should be relevant for monazite–(Ce), it is clear that the damage present in the natural samples is not equivalent to the sum of recoil damage and alpha-particle damage.

Whether or not alpha-assisted annealing is significant in natural monazite–(Ce), however, is still controversial. Deschanels et al. (2014) proposed this annealing mechanism as an explanation for their observation that Au-irradiated LaPO_4_ became amorphous whereas ^238^Pu-doped LaPO_4_ (alpha energy 5.59 MeV) remained crystalline at self-irradiation doses of up to 7.5 × 10^18^ α/g. Observations of highly self-irradiated phosphates that nevertheless have not experienced amorphisation have been documented before. For instance, Luo and Liu (2001) found LuPO_4_ doped with ^244^Cm (alpha energy 5.90 MeV) to be crystalline even after having sustained 50 × 10^18^ α/g. However, there are also apparently contrasting observations that monazite-structured phases may become amorphous even at much lower self-irradiation doses. For instance, Burakov et al. (2004) observed amorphisation of ^238^PuPO_4_ after having experienced only 0.86 × 10^18^ α/g, and Bregiroux et al. (2007) found ^241^AmPO_4_ (alpha energy 5.49 MeV) to be amorphised by 3.3 × 10^18^ α/g. Deschanels et al. (2014) attempted to assign the apparently contrasting examples above by the consideration that alpha annealing may only be effective at low damage-creation rates per time unit. This certainly still needs to be confirmed, and it needs to be clarified why low-rate recoil self-irradiation accompanied by the same low-rate helium self-irradiation should have a vastly different result compared to somewhat higher-rate recoil self-irradiation accompanied by the same higher-rate helium self-irradiation [note that the damage-creation rates effective for the crystalline sample of Luo and Liu (2001) and the amorphised sample of Bregiroux et al. (2007), expressed in dpa per s, differ by less than a factor of 2]. Apparently contrasting observations were also made in He-irradiation experiments. In a recent study, Seydoux-Guillaume et al. (2018) found that He irradiation may anneal amorphous LaPO_4_ whereas Nasdala et al. (2013b) found that He irradiation does create radiation damage in crystalline CePO_4_. More research is certainly needed to find out under which conditions and in which kind of material alpha particles create damage and/or heal existing damage, and how significant these processes are for the long-term structural behaviour of natural monazite–(Ce).

### Comparison with natural zircon samples

Our SRIM predictions for zircon, averaging 620–688 defects per alpha-decay event in this mineral (Table S6), are well below the estimated average of ca. 1000–2000 displacements per alpha-recoil cascade as proposed by Weber et al. (1994b), and they are very well below the estimate of ca. 5000 permanent displacements per alpha-decay event in zircon by Farnan et al. (2007). This may imply our data are underestimated. On the other hand, Stoller et al. (2013) have discussed that SRIM estimates of radiation damage tend to be too high if “detailed” calculations including full damage cascades are conducted (as performed in the present study). Instead, “quick” SRIM calculations not considering full damage cascades but following the model of Kinchin and Pease (1955), predict only about half as many displacements compared to results of “detailed” calculations. Stoller et al. (2013) found that these lower displacement numbers are widely consistent with predictions using the MARLOWE-based code and model of Norgett et al. (1975), and they suggested the “quick” Kinchin–Pease option be used in SRIM estimates of displacement numbers. The latter implies that, in contrast to the above, our SRIM-based estimates of atomic displacements created per alpha-decay event may still be overestimated. In view of the obvious uncertainties, our estimates are not considered as precise and reliable but they merely serve as a basis for roughly estimating the defect concentration in natural zircon, finally for providing a means of comparing irradiated and naturally damaged samples.

The dpa values calculated for natural samples (Table S7) are on the same order as dpa values of the Au-irradiated lamellae studied herein (Table 2). The two—internally consistent—trends of spectral changes versus dpa values, however, show a slight mismatch (Fig. 6b) that could be assigned to different causes. First, the assumption of Nasdala et al. (2001) that their four samples have not undergone any annealing since the time of closure of the U–Pb systems, may be wrong. For instance, the assumption that a Lunar zircon has spent most of its lifetime under “cold” conditions and hence must reflect (close to) complete retention of the radiation damage, was questioned by different Raman-FWHM versus alpha-dose relationships observed from other Lunar zircon samples (Pidgeon et al. 2015; Blum et al. 2017). More investigations of reliably non-annealed zircon samples of different ages will be needed to rule out this uncertainty. Second, it needs to be considered cautiously as to which degree Au irradiation at − 196 °C is fully comparable with the self-irradiation of natural samples, which occurs at clearly higher temperatures and hence may involve a higher fraction of immediate defect recombination. These doubts seem, however, contradicted by the observation of Nasdala et al. (2011) that similar degrees of damage were created in (otherwise identical) He-irradiation experiments at − 196 °C and 23 °C. This implies that immediate recombination at room temperature [perhaps in contrast to monazite–(Ce); cf. discussion above] may still be insignificant in the case of zircon. Third, our lamellae have only experienced heavy-ion irradiation. In future research, combined heavy- and light-ion irradiation should be done, in order to have more appropriate experimental equivalents of self-irradiation (i.e. alpha-decay event) damage. Fourth, Au irradiation may not be considered as a sufficiently precise equivalent of alpha-recoil damage, because of the different ion energies (1–10 MeV versus 0.06–0.16 MeV) and hence different fractions of nuclear and electronic stopping powers.

## Concluding remarks

In spite of the uncertainties discussed above, both Raman and PL spectroscopy are found to be promising tools for the in situ quantification of radiation damage in monazite–(Ce) and zircon. For typical monazite–(Ce), results are somewhat less precise, as Raman and PL spectroscopic parameters of this mineral often are significantly affected by elevated concentrations of non-formula elements (Ruschel et al. 2012). This of course also applies to rare zircon whose chemical compositions deviate strongly from the theoretical formula (e.g. Geisler et al. 2005; Zamyatin et al. 2017; Kudryashov et al. 2017). Nevertheless, with due consideration of possible effects of the chemical composition on spectra, Raman-band and PL-line broadening allows to quantify damage that is present in monazite–(Ce) and zircon. In both minerals, minor to severe radiation damage is characterised by defect concentrations in the approximate range 0.001–0.1 dpa. These present defect concentrations, however, often underestimate [in the case of monazite–(Ce) even by two orders of magnitude] the total self-irradiation experienced by a given natural sample. Furthermore, heavy-ion irradiation experiments at liquid-N_2_ temperature, as performed in the present study, open up the opportunity to study and compare directly the radiation tolerance of solids. Here, the accumulation of structural damage upon ion irradiation and resulting changes of spectroscopic parameters were found to be surprisingly similar in ZrSiO_4_ and CePO_4_ (compare Figs. 4, 5); both of the studied minerals experiences complete amorphisation at Au fluences of 4.5–12 × 10^13^ ions/cm^2^. These observations contrast again the assumption (Picot et al. 2008) that monazite-group minerals are more radiation-resistant than zircon and, hence, a more suited nuclear-waste form for the immobilisation of actinides and other radionuclides. The build-up of structural damage on the one hand, and immediate and/or post-damage annealing effects on the other hand [the latter obviously being much more effective in monazite–(Ce) at lower temperatures], always need to be considered as two separate processes.

## Electronic supplementary material

Below is the link to the electronic supplementary material.

Supplementary material 1 (PDF 377 KB)
