# Supplementary material for: Irradiation effects in monazite–(Ce) and zircon: Raman and photoluminescence study of Au-irradiated FIB foils
Source: Phys Chem Miner. 2018 May 23;45(9):855–71. doi: 10.1007/s00269-018-0975-9 (PMC6153653; doi:10.1007/s00269-018-0975-9)
Supplement: Supplementary file 1 — Supplementary material 1 (PDF 377 KB) [file 269_2018_975_MOESM1_ESM.pdf]

Supplementary material accompanying the article

Irradiation effects in monazite–(Ce) and zircon: Raman and photoluminescence study of Au-irradiated FIB foils

Lutz Nasdala • Shavkat Akhmadaliev • Andreas Artac • Chutimun Chanmuang N. • Gerlinde Habler • Christoph Lenz

Content:

1. General description of the samples studied
2. Ages of samples
3. Structural state and annealing
4. Calculation of defect densities (dpa) for natural monazite–(Ce) and zircon samples
Supplementary Figs. S1 and S2
Supplementary Tables S1–S7
References

---

1. General description of the samples studied

Sample R–5 was a clear transparent, light cognac-brown, seemingly un-zoned zircon crystal with rounded edges (~18 mm longest dimension), found in a gem-placer deposit in the Ban Lung area, Ratanakiri, Cambodia (see Smith and Balmer 2009). Sample Nd3 was a lemon-yellow to light pale-greenish crystal (~0.8 mm size) of short prismatic to thick tabular shape. This $CePO_4$ crystal (doped with a minor amount of Nd^{3+}) was grown using a $NaPO_3$ flux technique (for details see Lenz et al. 2013, 2015). Sample GM2 was a brownish yellow fragment (~2 mm size) of a monazite–(Ce) crystal from Itambé, Brazil. This sample has already been described in detail elsewhere (Ruschel et al. 2012). Sample N22 was a large (~20 mm longest dimension) monazite–(Ce) crystal from Madagascar, provided from the mineral collection of the Institute of Mineralogy, TU Bergakademie Freiberg, Germany.

2. Ages of samples

The age of monazite–(Ce) sample N22 (Madagascar) was determined by secondary ion mass spectrometry (SIMS) analysis, using the SHRIMP II system at the Department of Applied Physics, Curtin University of Technology, Perth. The monazite surface was sputtered with a primary, mass-filtered $(O_2)^-$ beam with ∼1 nA current, focused to a ∼7–10 µm spot. Mass resolution (M/ΔM) was better than 5000. The sensitivity for Pb isotopes was about 20 counts per second per ppm, per nA. A single analysis consisted of seven scans. Data for each spot were collected in sets of seven scans through the mass range of $^{202}LaPO_2$, $^{203}CePO_2$, ^{204}Pb, background near ^{204}Pb, ^{206}Pb, ^{207}Pb, ^{208}Pb, ^{232}Th, ^{238}U, $^{248}ThO_2$, and $^{270}UO_2$. The total analytical time was ca. 16 min per spot. Results obtained were calibrated against MAD–1, a 514 Ma old reference monazite (Kinny 1997). The ^{204}Pb method was used to correct the results for non-radiogenic Pb. For further analytical details, and details of the

data-reduction procedure, see Foster et al. (2000). Results obtained are shown in Supplementary Table S1, and data are plotted in a Concordia diagram in Fig. S1. Sample N22 yielded a mean $^{206}Pb/^{238}U$ age of 483±13 Ma and a mean $^{207}Pb/^{206}Pb$ age of 503±12 Ma (n = 6). These values correspond reasonably well with a mean U–Th–Pb age of 485±8 Ma (n = 7) that was calculated from the electron probe micro-analyser (EPMA) results, following the method of Montel et al. (1996; see also Cocherie and Albarede 2001).

The age of zircon sample R–5 (Ratanakiri, Cambodia) was determined for three fragments by means of solution isotope dilution thermal ionization mass spectrometry (ID–TIMS) analysis (Krogh 1973) at the Department of Geosciences, University of Oslo. Fragments were first cleaned with HNO_3, H_2O and acetone. After being weighed on a microbalance, fragments were transferred to Savillex vials and dissolved in HF after adding a ^{202}Pb–^{205}Pb–^{235}U spike. The Pb and U isotope compositions were then measured in a MAT262 mass spectrometer. The spike composition was calibrated against the synthetic ET100 solution (Condon et al. 2008), which is provided by the EARTHTIME initiative (http://www.earthtime.org). For further details of the sample-solution and isotope measurement procedures, the reader is referred to Corfu (2004). All isotope ratios and ages were corrected for fractionation, spike, blank ($^{206}Pb/^{204}Pb$ = 18.3; $^{207}Pb/^{204}Pb$ = 15.555) and initial common Pb (based on Stacey and Kramers 1975). Errors were calculated by propagating the main sources of uncertainty. The U–Pb ratio of the spike used is adapted to $^{206}Pb/^{238}U$ = 0.015660 for the ET100 solution, as obtained with the ET2535 spike at the NERC Isotope Geosciences Laboratory. Results are presented in Supplementary Table S2, and data are plotted in a Concordia diagram in Fig. S2. The very young Ratanakiri zircon yielded a mean $^{206}Pb/^{238}U$ age of 0.92±0.07 Ma (n = 7).

A ^{238}U decay constant of 1.55125×10^{-10} a^{-1} and a ^{235}U decay constant of 9.84850×10^{-10} a^{-1} (Jaffey et al. 1971) were used for the conversion of isotopic ratios into ages, and for plotting the results. For the calculation of ages and for the preparation of Concordia plots, the Isoplot program (Ludwig 2003) was used.

Monazite–(Ce) sample GM2 (Itambé, Brazil) was not subjected to age determination in the present study. According to Ruschel et al. (2012), this sample has a TIMS-determined age of 493±6 Ma.

3. Structural state and annealing

Unit-cell parameters of the raw (i.e. unannealed) samples and their heat-treated analogues were determined by means of single crystal X-ray diffraction. A Huber 5042 four-circle diffractometer with point detector, horizontal and vertical diffracted beam slits, and un-monochromatized Mo radiation was used. Operating conditions were 50 kV and 32 mA. Constrained lattice parameters were refined from the positions of approximately 30 Bragg reflections for each crystal. Corrections were carried out by applying the method of eight-position diffracted-beam centering (King and Finger 1979), which allows one to eliminate errors in peak positions due to sample displacement (Ullrich et al. 2009). Results are shown in Supplementary Table S3, along with selected spectroscopic parameters.

4. Calculation of defect densities (dpa) for natural monazite–(Ce) and zircon samples

In order to get a means for comparing (semi-) quantitatively the irradiation-induced damage in our FIB lamellae on the one hand with the self-irradiation damage in natural monazite–(Ce) and zircon samples on the other hand, we needed to calculate average defect densities for all samples. Following what is commonplace in the literature, we use the average of atomic displacements per lattice atom (dpa) to express defect densities.

For the calculation of dpa values for naturaly self-irradiated monazite–(Ce) and zircon samples (whose radiation damage is assigned predominantly to alpha decays), we have first predicted the number of atomic

displacements created per alpha event in $CeOP_4$ and $ZrSiO_4$. Calculations were done using the SRIM (the Stopping and Range of Ions in Matter; Ziegler et al. 1985; 2010) computer code, version SRIM–2012.03. For $CePO_4$, the target density was assumed to be 7.915×10^{22} atoms/cm^3, or 5.15 g/cm^3; and SRIM defaults for displacement energies (Ce, 25 eV; P, 25 eV; O 28 eV) were accepted. For $ZrSiO_4$, the target density was assumed to be 9.165×10^{22} atoms/cm^3, or 4.65 g/cm^3; and displacement energies of Moreira et al. (2009: Zr, 75 eV; Si 75 eV; O, 60 eV) were used. To reach reliable statistics, 3000 incoming ions were calculated. Calculations were done for all alpha energies in the ^{238}U, ^{235}U and ^{232}Th decay series that have a relative probability of ≥10 % in the particular decay event (compare Firestone and Shirley 1996). Calculated displacements as created by the alpha particle itself and by the recoiled nucleus were added up, and a weighted mean number of displacements per alpha-event (considering the relative probabilities of the individual energies) was calculated for the respective decay series. Results are shown in Supplementary Tables S4 ($CePO_4$) and S6 ($ZrSiO_4$), respectively.

These means were used to convert time-integrated alpha doses of previously studied natural monazite–(Ce) and zircon samples into damage concentrations. Using the respective mean displacement number per event, the ^{238}U, ^{235}U and ^{232}Th fluxes (events per g) were converted to displacement numbers (per g). Monazite–(Ce) has a unit-cell volume of ~300 Å^3, which converts to ~3.33×10^{21} unit cells per cm^3. As the unit cell of monazite–(Ce) (Z=4) contains 24 atoms, the former value converts to ~8×10^{22} atoms per cm^3. This in turn converts to 15.534×10^{21} atoms per g (assuming specific gravity of 5.15 g/cm^3). The dpa value was then calculated by dividing the calculated displacements number (per g) by 15.534×10^{21} atoms (per g). Results are quoted in Supplementary Table S5. The analogous procedure was used for zircon. This mineral (Z = 4) has a unit-cell volume of ~260 Å^3, which converts to ~3.846×10^{21} unit cells per cm^3 and ~9.23×10^{22} atoms per cm^3. This in turn converts to 19.851×10^{21} atoms per g (assuming specific gravity of 4.65 g/cm^3). The dpa values calculated for the “unannealed” natural zircon samples of Nasdala et al. (2001) are quoted in Supplementary Table S7.

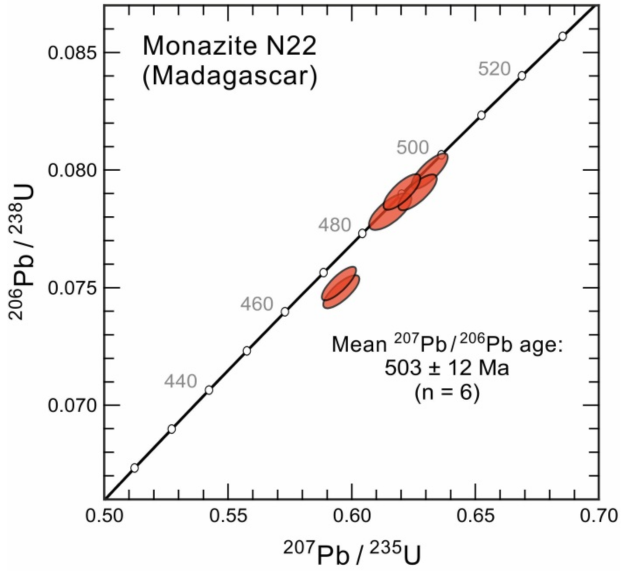

Fig. S1 Concordia plot of U-Pb data for monatite–(Ce) sample N22. Error ellipses represent 2σ uncertainties

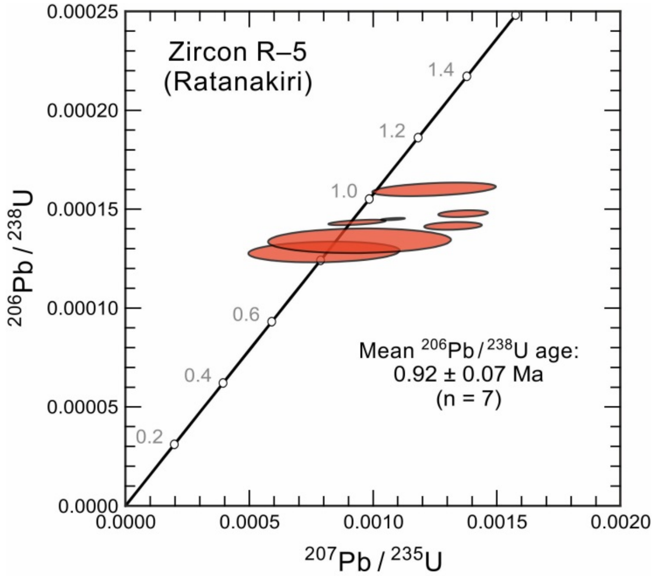

Fig. S2 Concordia plot of U-Pb data for zircon sample R–5. Error ellipses represent 2σ uncertainties

Supplementary Table S1 SHRIMP results for monazite–(Ce) N22 (Madagascar)

Spot	f 206* [%]	$^{206}Pb/^{238}U$	$^{206}Pb/^{238}U$ age [Ma]	$^{207}Pb/^{235}U$	$^{207}Pb/^{235}U$ age [Ma]	$^{207}Pb/^{206}Pb$	$^{207}Pb/^{206}Pb$ age [Ma]	Disc.** [%]
1	0.19	0.07905±0.00062	490±4	0.6266±0.0064	494±4	0.05749±0.00032	510±12	4
2	0.12	0.07517±0.00058	467±4	0.5948±0.0056	474±4	0.05739±0.00026	507±10	8
3	0.21	0.07907±0.00061	491±4	0.6202±0.0061	490±4	0.05689±0.00029	502±11	–1
4	0.18	0.07995±0.00062	496±4	0.6314±0.0061	497±4	0.05728±0.00028	494±15	1
5	0.20	0.07824±0.00064	486±4	0.6156±0.0070	487±4	0.05706±0.00039	494±15	2
6	0.05	0.07483±0.00057	465±3	0.5958±0.0059	475±4	0.05775±0.00032	520±12	11

Note: Isotope ratios and ages are corrected for common Pb (^{204}Pb method)
* f206 = common ^{206}Pb / total ^{206}Pb, from the observed ^{204}Pb
** Disc. = U–Pb discordance: 100 % minus the ratio of the ^{204}Pb–corrected $^{206}Pb/^{238}U$ and $^{207}Pb/^{206}Pb$ ages

Supplementary Table S2 ID–TIMS results for zircon R–5 (Ratanakiri, Cambodia)

Analysis no.	Weight [μg]	Pb_{com}* [pg]	$^{206}Pb/^{204}Pb$**	$^{206}Pb/^{238}U$	$^{206}Pb/^{238}U$ age [Ma]	$^{207}Pb/^{206}Pb$	$^{207}Pb/^{206}Pb$
389/10	529	9.8	37.8	0.000160±0.000003	1.03±0.02	0.00124±0.00021	0.0563±0.0100
389/10***	–	3.6	64.9	0.000143±0.000001	0.92±0.01	0.00093±0.00010	0.0472±0.0051
391/1	1166	1.8	191.1	0.000145±0.000000	0.93±0.00	0.00108±0.00004	0.0539±0.0020
394/1	585	16.4	29.0	0.000128±0.000004	0.83±0.03	0.00080±0.00025	0.0451±0.0155
391/2	840	8.5	56.6	0.000148±0.000001	0.95±0.01	0.00136±0.00008	0.0668±0.0044
394/2	948	14.9	47.9	0.000142±0.000002	0.91±0.01	0.00132±0.00010	0.0676±0.0054
394/3	833	29.1	27.1	0.000134±0.000005	0.86±0.03	0.00094±0.00030	0.0510±0.0187

Note: The $^{206}Pb/^{238}U$ isotope ratios and ages were corrected for ^{230}Th disequilibrium according to Schärer (1984) and assuming Th/U magma = 4
* Pb_{com} = total common Pb in sample (initial + blank)
** Raw data, corrected for fractionation and spike
*** The solution for 389/10 was separated after dissolution and before chemical separation, for aliquot analysis

Supplementary Table S3 Unit-cell constants and selected spectroscopic parameters of unirradiated samples

Sample*	X-ray diffraction					Raman		PL	
								Dy^{3+}	Nd^{3+}
	a [Å]	b [Å]	c [Å]	β [°]	V [$Å^3$]	FWHM [cm^{-1}]	Shift [cm^{-1}]	FWHM [cm^{-1}]	FWHM [cm^{-1}]
Monazite–(Ce):									
Nd3	6.7953(5)	7.0230(3)	6.4697(3)	103.454(4)	300.29(3)	2.6±0.3	970.4±1.0	–	29±3
N22 raw	6.771(2)	6.986(2)	6.476(2)	103.92(2)	297.4(2)	19.2±1.5	972.9±0.9	–	104±12
N22 ann.	6.7528(5)	6.9693(3)	6.4392(4)	103.734(4)	294.38(3)	7.3±1.0	976.0±0.7	–	63±6
GM2 raw	6.4737(7)	6.9958(7)	6.8061(6)	103.595(8)	299.60(5)	14.0±1.0	972.2±0.7	–	82±9
GM2 ann.	6.7603(5)	6.9812(3)	6.4419(4)	103.572(4)	295.54(3)	6.9±0.5	975.3±0.6	–	53±5
Reference_1	6.7880(10)	7.0163(8)	6.4650(7)	103.53(1)	299.49(13)				
Reference_2						2.3±0.4	970.4±0.5		
Zircon:									
R–5 raw	6.6037(1)	–	5.9789(2)	–	260.73(1)	1.8±0.2	1007.6±0.5	13±1	15±2
R–5 ann	6.6038(1)	–	5.9791(1)	–	260.75(1)	1.8±0.2	1007.6±0.5	13±1	15±2
Reference_3	6.606(1)	–	5.977(1)	–	260.82(5)				
Reference_4						1.8±0.2	1008.3±0.5		

Note: All values quoted are means of multiple analyses

* raw = unannealed samples; ann. = annealed samples

Reference_1: Unit-cell parameters of synthetic $CePO_4$ from Ni et al. (1995)

Reference_2: Raman data of synthetic $CePO_4$ from Ruschel et al. (2012)

Reference_3: Unit-cell parameters of synthetic $ZrSiO_4$ from von Westrenen et al. (2004)

Reference_4: Raman data of synthetic $ZrSiO_4$ from Nasdala et al. (2002)

PL = photoluminescence

FWHM = full width at half maximum

Monazite–(Ce) samples: Spectral parameters are quoted for the $\nu_1(PO_4)$ Raman band and the 11600 cm^{-1} sublevel of the $^4F_{3/2} \rightarrow {}^4I_{9/2}$ emission of Nd^{3+}

Zircon samples: Spectral parameters are quoted for the $\nu_3(SiO_4)$ Raman band, the 11360 cm^{-1} sublevel of the $^4F_{3/2} \rightarrow {}^4I_{9/2}$ emission of Nd^{3+}, and for the 17210 cm^{-1} sublevel of the $^4F_{9/2} \rightarrow {}^4H_{13/2}$ emission of Dy^{3+}

Supplementary Table S4 Results of Monte Carlo simulations (SRIM) of alpha-event effects in monazite–(Ce)

Number in decay chain	Alpha-decay event	Probability* [%]	Alpha particle Energy [MeV]*	Displ.	Alpha recoil Energy [MeV]*	Displ.	Total displacements
^{238}U decay chain:							
1	$^{238}U \rightarrow {}^{234}Th$	79	4198	172	71.8	1003	1175
		21	4151	170	71.0	992	1162
2	$^{234}U \rightarrow {}^{230}Th$	71	4775	180	83.0	1144	1324
		29	4722	181	82.2	1134	1315
3	$^{230}Th \rightarrow {}^{226}Ra$	76	4688	177	83.0	1134	1311
		23	4621	179	81.8	1119	1298
4	$^{226}Ra \rightarrow {}^{222}Rn$	99	4784	178	86.2	1170	1348
5	$^{222}Rn \rightarrow {}^{218}Po$	100	5490	180	100.7	1341	1521
6	$^{218}Po \rightarrow {}^{214}Pb$	100	5304	184	103.0	1379	1563
7	$^{214}Po \rightarrow {}^{210}Pb$	100	7687	202	146.5	1887	2089
8	$^{210}Po \rightarrow {}^{206}Pb$	100	5304	184	103.0	1372	1556
			Weighted mean per alpha event in ^{238}U decay chain:				1484
^{235}U decay chain:							
1	$^{235}U \rightarrow {}^{231}Th$	55	4398	174	76.2	1059	1233
		17	4366	177	75.5	1050	1227
2	$^{231}Pa \rightarrow {}^{227}Ac$	25	5014	180	88.4	1208	1388
		23	4951	178	87.3	1195	1373
		20	5028	183	88.7	1212	1395
		11	5059	183	89.2	1218	1401
3	$^{227}Th \rightarrow {}^{223}Ra$	24	6038	185	108.4	1436	1621
		24	5978	185	107.3	1424	1609
		20	5757	181	103.3	1375	1556
4	$^{223}Ra \rightarrow {}^{219}Rn$	53	5716	183	104.5	1389	1572
		26	5607	189	102.5	1363	1552
5	$^{219}Rn \rightarrow {}^{215}Po$	79	6819	195	126.9	1650	1845
		13	6553	194	122.0	1591	1785
6	$^{215}Po \rightarrow {}^{211}Pb$	100	7386	198	140.0	1810	2008
7	$^{211}Bi \rightarrow {}^{207}Tl$	84	6623	193	128.0	1658	1851
		16	6278	192	121.4	1582	1774
			Weighted mean per alpha event in ^{235}U decay chain:				1597
^{232}Th decay chain:							
1	$^{232}Th \rightarrow {}^{228}Ra$	78	4013	166	70.4	976	1142
		22	3954	169	69.6	966	1135
2	$^{228}Th \rightarrow {}^{224}Ra$	71	5423	182	96.9	1298	1480
		28	5340	180	95.4	1281	1461
3	$^{224}Ra \rightarrow {}^{220}Rn$	95	5685	183	103.4	1377	1560
4	$^{220}Rn \rightarrow {}^{216}Po$	100	6288	187	116.5	1526	1713
5	$^{216}Po \rightarrow {}^{212}Pb$	100	6778	195	127.9	1672	1867
6a (64.06 %)	$^{212}Po \rightarrow {}^{208}Pb$	100	8784	207	169.0	2139	2346
6b (35.94 %)	$^{212}Bi \rightarrow {}^{208}Tl$	70	6051	184	116.4	1522	1706
		27	6090	188	117.2	1532	1720
			Weighted mean per alpha event in ^{232}Th decay chain:				1645

* Data extracted from Firestone and Shirley (1996)
Displ. = number of atomic displacements calculated

Supplementary Table S5 Calculation of defect densities (dpa) for natural monazite–(Ce) samples

Sample	Age [Ma]	UO_2 [wt%]	ThO_2 [wt%]	Alpha doses [×10^{18} / g]* ^{238}U	^{235}U	^{232}Th	Total	Displacements [×10^{21} / g]** ^{238}U	^{235}U	^{232}Th	Total	Dpa
Burnet	1096	0.22	8.26	7.2	0.5	63.0	70.7	10.7	0.8	103.6	115.1	7.41
D. Bory	337	4.85	1.33	46.1	2.2	3.1	51.3	68.4	3.5	5.0	76.9	4.95
ECA6	369	0.32	5.35	3.3	0.2	13.5	17.0	5.0	0.3	22.2	27.4	1.76
Elk Mountain	1391	0.28	9.76	11.9	0.9	95.1	108.0	17.7	1.5	156.5	175.7	11.31
F6	560	0.13	11.3	2.1	0.1	43.4	45.6	3.1	0.2	71.5	74.7	4.81
GM2	493	0.68	6.57	9.6	0.5	22.2	32.2	14.2	0.8	36.5	51.5	3.31
GM3	484	0.55	7.76	7.6	0.4	25.7	33.7	11.3	0.6	42.3	54.2	3.49
JUP	101	1.17	13.2	3.3	0.1	9.0	12.5	4.9	0.2	14.9	20.0	1.28
Madmon	496	0.43	11.4	6.1	0.3	38.8	45.1	9.0	0.5	63.7	73.3	4.72
Moacyr	506	0.08	6.19	1.2	0.1	21.5	22.7	1.7	0.1	35.3	37.1	2.39
Moacir	474	0.11	6.51	1.5	0.1	21.1	22.7	2.2	0.1	34.8	37.1	2.39
MOM3	450	0.11	6.47	1.4	0.1	19.9	21.4	2.1	0.1	32.8	35.0	2.25
Namon	485	0.29	10.1	4.0	0.2	33.6	37.8	6.0	0.3	55.2	61.5	3.96
SIV	507	0.24	4.23	3.5	0.2	14.7	18.4	5.2	0.3	24.2	29.6	1.91
SL	491	0.46	19.6	6.4	0.3	65.9	72.7	9.6	0.5	108.5	118.6	7.63
VK-1	488	0.82	14.2	11.4	0.6	47.5	59.5	16.9	0.9	78.1	96.0	6.18
N22	503	0.47	10.8	6.7	0.3	37.2	44.3	10.0	0.5	61.3	71.8	4.62

Note: Data for all samples except N22 (investigated in the present study) are from Ruschel et al. (2012)

* Alpha doses were calculated from ages and present U and Th concentrations according to Holland and Gottfried (1955) and Murakami et al. (1991)

** Calculated as the products of alpha doses and the weighted mean number of displacements per alpha event in the respective decay chain (from Supplementary Table S4)

Dpa = average of atomic displacements per lattice atom

Supplementary Table S6 Results of Monte Carlo simulations (SRIM) of alpha-event effects in zircon

Number in decay chain	Alpha-decay event	Probabi-lity* [%]	Alpha particle Energy [MeV]*	Alpha particle Displ.	Alpha recoil Energy [MeV]*	Alpha recoil Displ.	Total displace-ments
^{238}U decay chain:							
1	$^{238}U \rightarrow ^{234}Th$	79	4198	67	71.8	425	492
		21	4151	66	71.0	421	487
2	$^{234}U \rightarrow ^{230}Th$	71	4775	65	83.0	485	550
		29	4722	69	82.2	480	549
3	$^{230}Th \rightarrow ^{226}Ra$	76	4688	68	83.0	480	548
		23	4621	68	81.8	474	542
4	$^{226}Ra \rightarrow ^{222}Rn$	99	4784	66	86.2	495	561
5	$^{222}Rn \rightarrow ^{218}Po$	100	5490	72	100.7	565	637
6	$^{218}Po \rightarrow ^{214}Pb$	100	5304	72	103.0	580	652
7	$^{214}Po \rightarrow ^{210}Pb$	100	7687	77	146.5	795	872
8	$^{210}Po \rightarrow ^{206}Pb$	100	5304	72	103.0	580	652
			Weighted mean per alpha event in ^{238}U decay chain:				620
^{235}U decay chain:							
1	$^{235}U \rightarrow ^{231}Th$	55	4398	68	76.2	448	516
		17	4366	68	75.5	444	512
2	$^{231}Pa \rightarrow ^{227}Ac$	25	5014	68	88.4	511	579
		23	4951	69	87.3	505	574
		20	5028	69	88.7	512	581
		11	5059	69	89.2	515	584
3	$^{227}Th \rightarrow ^{223}Ra$	24	6038	71	108.4	609	680
		24	5978	72	107.3	602	674
		20	5757	71	103.3	584	655
4	$^{223}Ra \rightarrow ^{219}Rn$	53	5716	71	104.5	588	659
		26	5607	72	102.5	578	650
5	$^{219}Rn \rightarrow ^{215}Po$	79	6819	74	126.9	695	769
		13	6553	76	122.0	670	746
6	$^{215}Po \rightarrow ^{211}Pb$	100	7386	74	140.0	763	837
7	$^{211}Bi \rightarrow ^{207}Tl$	84	6623	72	128.0	700	772
		16	6278	72	121.4	668	740
			Weighted mean per alpha event in ^{235}U decay chain:				684
^{232}Th decay chain:							
1	$^{232}Th \rightarrow ^{228}Ra$	78	4013	65	70.4	413	478
		22	3954	65	69.6	409	474
2	$^{228}Th \rightarrow ^{224}Ra$	71	5423	71	96.9	550	621
		28	5340	71	95.4	543	614
3	$^{224}Ra \rightarrow ^{220}Rn$	95	5685	72	103.4	582	654
4	$^{220}Rn \rightarrow ^{216}Po$	100	6288	72	116.5	644	716
5	$^{216}Po \rightarrow ^{212}Pb$	100	6778	73	127.9	704	777
6a (64.06 %)	$^{212}Po \rightarrow ^{208}Pb$	100	8784	79	169.0	901	980
6b (35.94 %)	$^{212}Bi \rightarrow ^{208}Tl$	70	6051	71	116.4	644	715
		27	6090	73	117.2	648	721
			Weighted mean per alpha event in ^{232}Th decay chain:				688

* Data extracted from Firestone and Shirley (1996)
Displ. = number of atomic displacements calculated

Supplementary Table S7 Calculation of defect densities (dpa) for natural zircon samples

Sample	Age [Ma]	U [ppm]	Th [ppm]	Alpha doses [×10^{18} / g]* ^{238}U	^{235}U	^{232}Th	Total	Displacements [×10^{19} / g]** ^{238}U	^{235}U	^{232}Th	Total	Dpa
Zircon grains from Saxonian rhyolites (data from Nasdala et al. 1998):												
64-2/A	278	197	103	0.17	0.01	0.02	0.20	10.8	0.5	1.5	12.9	0.0065
64-2/B	278	414	332	0.37	0.02	0.07	0.45	22.7	1.2	4.9	28.8	0.0145
64-2/F	278	66	31	0.06	0.00	0.01	0.07	3.6	0.2	0.5	4.3	0.0021
4-2/C	278	270	138	0.24	0.01	0.03	0.28	14.8	0.8	2.0	17.6	0.0089
4-2/D	278	672	424	0.59	0.03	0.09	0.71	36.9	1.9	6.3	45.0	0.0227
4-3/A	278	278	190	0.25	0.01	0.04	0.30	15.2	0.8	2.8	18.8	0.0095
4-3/B	278	211	148	0.19	0.01	0.03	0.23	11.6	0.6	2.2	14.4	0.0072
Lunar zircon (data from Wopenka et al. 1996):												
A	4000	21	6	0.36	0.14	0.02	0.52	22.5	9.3	1.4	33.2	0.0167
B	4000	31	11	0.53	0.20	0.04	0.77	33.2	13.8	2.6	49.5	0.0250
C	4000	44	21	0.76	0.29	0.07	1.12	47.1	19.6	4.9	71.6	0.0361
D	4000	51	29	0.88	0.33	0.10	1.31	54.6	22.7	6.8	84.1	0.0423
E	4000	55	31	0.95	0.36	0.11	1.41	58.8	24.5	7.3	90.6	0.0456
Rims of zircon crystals from two Saxonian monzonites (data from Nasdala et al.1999) :												
Leu-2/1	326	942	447	0.98	0.05	0.11	1.14	60.8	3.1	7.8	71.8	0.0363
Leu-2/2	326	732	428	0.76	0.04	0.11	0.91	47.3	2.4	7.5	57.2	0.0288
Leu-2/3	326	706	412	0.74	0.03	0.10	0.87	45.6	2.4	7.2	55.1	0.0278
Leu-2/4	326	1426	910	1.48	0.07	0.23	1.79	92.1	4.8	15.8	112.7	0.0568
Leu-2/5	326	619	144	0.64	0.03	0.04	0.71	40.0	2.1	2.5	44.5	0.0224
Leu-3/1	326	517	295	0.54	0.03	0.07	0.64	33.4	1.7	5.1	40.2	0.0203
Hei-1/1	330	695	342	0.73	0.03	0.09	0.85	45.4	2.4	6.0	53.8	0.0271
Hei-1/2	330	686	425	0.72	0.03	0.11	0.87	44.8	2.3	7.5	54.7	0.0275
Hei-1/3	330	425	336	0.45	0.02	0.09	0.56	27.8	1.4	5.9	35.2	0.0177
Hei-1/4	330	352	276	0.37	0.02	0.07	0.46	23.0	1.2	4.9	29.1	0.0146
Hei-1/5	330	378	292	0.40	0.02	0.07	0.49	24.7	1.3	5.1	31.1	0.0157
Hei-2/1	330	277	236	0.29	0.01	0.06	0.37	18.1	0.9	4.2	23.2	0.0117
Hei-3/1	330	634	346	0.67	0.03	0.09	0.79	41.4	2.2	6.1	49.7	0.0250
Hei-4/1	330	721	339	0.76	0.04	0.09	0.88	47.1	2.4	6.0	55.6	0.0280
Zircon grains from the Frankenstein gabbro, Odenwald (data from Nasdala et al. 2001):												
Fr-1/a	362	441	202	0.51	0.02	0.06	0.59	31.7	1.7	3.9	37.3	0.0189
Fr-1/b	362	379	158	0.44	0.02	0.04	0.50	27.2	1.4	3.1	31.7	0.0160
Fr-1/c	362	1115	808	1.29	0.06	0.23	1.58	80.2	4.2	15.6	100.0	0.0504
Fr-2/a	362	379	220	0.44	0.02	0.06	0.52	27.2	1.4	4.3	32.9	0.0166
Fr-2/b	362	494	343	0.57	0.03	0.10	0.70	35.5	1.9	6.6	44.0	0.0222
Fr-2/c	362	132	40	0.15	0.01	0.01	0.17	9.5	0.5	0.8	10.8	0.0054
Fr-3	362	229	40	0.27	0.01	0.01	0.29	16.5	0.9	0.8	18.1	0.0091

Note: The above four zircon populations were proposed as “unannealed” by Nasdala et al. (2001); samples are assumed to have stored (nearly) completely the radiation damage since the time of primary growth

* Alpha doses were calculated from ages and present U and Th concentrations according to Holland and Gottfried (1955) and Murakami et al. (1991)

** Calculated as the products of alpha doses and the weighted mean number of displacements per alpha event in the respective decay chain (from Supplementary Table S6)

Dpa = average of atomic displacements per lattice atom

References

Cocherie A, Albarede F (2001) An improved U-Th-Pb age calculation for electron microprobe dating of monazite. Geochim Cosmochim Ac 65:4509–4522

Condon DJ, McLean NM, Schoene B, Bowring SA, Parrish RR, Noble S (2008) Synthetic U-Pb 'standard' solutions for ID-TIMS geochronology. Geochim Cosmochim Ac 72(12S):A175

Corfu F (2004) U-Pb age, setting, and tectonic significance of the anorthosite-mangerite-charnockite-granite-suite, Lofoten-Vesterålen, Norway. J Petrol 45:1799–1819

Firestone RB, Shirley VS (1996) Table of isotopes, vol 2. John Wiley & Sons Inc

Foster G, Kinny P, Vance D, Prince C, Harris N (2000) The significance of monazite U-Th-Pb age data in metamorphic assemblages; a combined study of monazite and garnet chronometry. Earth Planet Sc Lett 181:327–340

Jaffey AH, Flynn KF, Glendenin LE, Bentley WC, Essling AM (1971) Precision measurement of half-lives and specific activities of ^{235}U and ^{238}U. Phys Rev C 4:1889–1906

Holland HD, Gottfried D (1955) The effect of nuclear radiation on the structure of zircon. Acta Crystallogr 8:291–300

King HE, Finger LW (1979) Diffracted beam crystal centering and its application to high-pressure crystallography. J Appl Crystallogr 12:374–378

Kinny P (1997) Users guide to U-Th-Pb dating of titanite, perovskite, monazite and baddeleyite using the W.A. SHRIMP. Curtin University of Technology, School of Physical Sciences Report SPS 693/1997/AP72

Krogh TE (1973) A low contamination method for the hydrothermal decomposition of zircon and extraction of U and Pb for isotopic age determinations. Geochim Cosmochim Ac 37:485–494

Lenz C, Talla D, Ruschel K, Škoda R, Götze J, Nasdala L (2013) Factors affecting the Nd^{3+} (REE^{3+}) luminescence in minerals. Miner Petrol 107:415–428

Lenz C, Nasdala L, Talla D, Hauzenberger C, Seitz R, Kolitsch U (2015) Laser-induced REE^{3+} photoluminescence of selected accessory minerals – An "advantageous artefact" in Raman spectroscopy. Chem Geol 415:1–16

Ludwig KR (2003) User's manual for Isoplot 3.00: A geochronological toolkit for Microsoft Excel. Berkeley Geochronology Center Special Publication, no 4, 71p

MonteI JM, Foret S, Veschambre M, Nicollet Ch, Provost A (1996) A fast, reliable, inexpensive insitu dating technique: electron microprobe ages on monazite. Chem Geol 131:37–53

Moreira PAFP, Devanathan R, Yu J, Weber WJ (2009) Molecular-dynamics simulation of threshold displacement energies in zircon. Nucl Instrum Meth B 267:3431–3436

Murakami T, Chakoumakos BC, Ewing RC, Lumpkin GR, Weber WJ (1991) Alpha-decay event damage in zircon. Am Mineral 76:1510–1532

Nasdala L, Götze J, Pidgeon RT, Kempe U, Seifert T (1998) Constraining a SHRIMP U-Pb age: micro-scale characterization of zircons from Saxonian Rotliegend rhyolites. Contrib Mineral Petr 132:300–306

Nasdala L, Wenzel T, Pidgeon RT, Kronz A (1999) Internal structures and dating of complex zircons from Meissen Massif monzonites, Saxony. Chem Geol 156:331-341

Nasdala L, Wenzel M, Vavra G, Irmer G, Wenzel T, Kober B (2001) Metamictisation of natural zircon: accumulation versus thermal annealing of radioactivity-induced damage. Contrib Mineral Petr 141:125–144

Nasdala L, Lengauer CL, Hanchar JM, Kronz A, Wirth R, Blanc P, Kennedy AK, Seydoux-Guillaume A-M (2002) Annealing radiation damage and the recovery of cathodoluminescence. Chem Geol 191:121–140

Ni Y, Hughes JM, Mariano AN (1995) Crystal chemistry of the monazite and xenotime structures. Am Mineral 80:21–26

Ruschel K, Nasdala L, Kronz A, Hanchar JM, Többens DM, Škoda R, Finger, F, Möller A (2012) A Raman spectroscopic study on the structural disorder of monazite–(Ce). Miner Petrol 105:41–55

Schärer U (1984) The effect of initial ^{230}Th disequilibrium on young U-Pb ages: the Makalu case, Himalaya. Earth Planet Sc Lett 67:191–204

Smith MH, Balmer WA (2009) Zircon mining in Cambodia. Gems Gemol 45:152–153

Stacey JS, Kramers JD (1975) Approximation of terrestrial lead isotope evolution by a two-stage model. Earth Planet Sc Lett 26:206–221

Ullrich A, Schranz W, Miletich R (2009) The nonlinear anomalous lattice elasticity associated with the high-pressure phase transition in spodumene: a high-precision static compression study. Phys Chem Miner 36:545–555

van Westrenen W, Frank MR, Hanchar JM, Fei Y, Finch RJ, Zha C-S (2004) In situ determination of the compressibility of synthetic pure zircon (ZrSiO4) and the onset of the zircon-reidite phase transition. Am Mineral 89:197–203

Wopenka B, Jolliff BL, Zinner E, Kremser DT (1996) Trace element zoning and incipient metamictization in a lunar zircon: application of three microprobe techniques. Am Mineral 81:902–912

Ziegler JF, Biersack JP, Littmark U (1985) The stopping and range of ions in solids. Pergamon, New York

Ziegler JF, Biersack JP, Littmark U (2010) SRIM – The stopping and range of ions in matter (2010). Nucl Instrum Meth B 268:1818–1823
